# Modeling energy balance while correcting for measurement error via free knot splines

**DOI:** 10.1371/journal.pone.0201892

**Published:** 2018-08-30

**Authors:** Daniel Ries, Alicia Carriquiry, Robin Shook

**Affiliations:** 1 Statistical Sciences Department, Sandia National Laboratories, Albuquerque, NM, United States of America; 2 Department of Statistics, Iowa State University, Ames, IA, United States of America; 3 Center for Children’s Healthy Lifestyles & Nutrition, Children’s Mercy, Kansas City, MO, United States of America; Central South University, CHINA

## Abstract

Measurements of energy balance components (energy intake, energy expenditure, changes in energy stores) are often plagued with measurement error. Doubly-labeled water can measure energy intake (EI) with negligible error, but is expensive and cumbersome. An alternative approach that is gaining popularity is to use the energy balance principle, by measuring energy expenditure (EE) and change in energy stores (ES) and then back-calculate EI. Gold standard methods for EE and ES exist and are known to give accurate measurements, albeit at a high cost. We propose a joint statistical model to assess the measurement error in cheaper, non-intrusive measures of EE and ES. We let the unknown true EE and ES for individuals be latent variables, and model them using a bivariate distribution. We try both a bivariate Normal as well as a Dirichlet Process Mixture Model, and compare the results via simulation. Our approach, is the first to account for the dependencies that exist in individuals’ daily EE and ES. We employ semiparametric regression with free knot splines for measurements with error, and linear components for error free covariates. We adopt a Bayesian approach to estimation and inference and use Reversible Jump Markov Chain Monte Carlo to generate draws from the posterior distribution. Based on the semiparameteric regression, we develop a calibration equation that adjusts a cheaper, less reliable estimate, closer to the true value. Along with this calibrated value, our method also gives credible intervals to assess uncertainty. A simulation study shows our calibration helps produce a more accurate estimate. Our approach compares favorably in terms of prediction to other commonly used models.

## Introduction

Obesity is perhaps the most serious public health problem of the 21st century, given the prevalence, global reach, and widespread health, economic, and social consequences. While the weight gain and lost is most certainly a complex interplay of a large number of factors across a variety of domains [1], ultimately a chronic energy surplus or deficit (energy intake versus energy expenditure) determines body weight change [2–6]. However, accurately measuring energy balance in free-living individuals is challenging, even in small studies. Yet to design effective public health policies and interventions, it would be valuable to be able to assess energy balance in nationwide surveys such as National Health and Nutrition Examination Survey (NHANES). Clearly, instruments such as doubly labeled water (DLW) and dual-energy X-ray absorptiometry (DXA) are too costly and burdensome to administer in large groups. Alternatively, consumer devices designed to measure physical activity and body composition are generally affordable, easy to use, and popular (an estimated 45 million will be sold in 2017), [7] but have varying levels of validity and reliability [8–10].

Within the past decade mathematical models have been formulated based on the principles of the first law of thermodynamics (rate of energy storage = rate of energy intake—rate of energy expenditure) [11]. Developed with multiple datasets containing gold-standard measures of energy expenditure, energy intake, and changes in energy storage (e.g. body composition using a two-compartment model of fat mass and fat-free mass) during periods of overfeeding [12] or caloric restriction [13], researchers have developed and refined a model based on the energy balance principle [14–16]. The result is a simple, easy-to-use equation that offers great promise in the quest for estimating energy intake using objectively measured methods. We have recently used these energy balance equations to compare estimates of energy intake obtained through gold-standard methods (DLW) and arm-based activity monitors (Sensewear Armband, BodyMedia Inc. Pittsburgh, PA) [17]. We observed very low group error in the estimates of energy expenditure and equation-derived energy intake using both the DLW and armband, indicating equivalency between the measures. However, the individual error for equation-derived energy intake and expenditure was quite large, likely due to large individual measurement error.

Therefore, a question of interest is whether measurements of energy balance obtained from self-report instruments or even from objective measuring tools such as the Sensewear Armband or other consumer devices, which are much less costly to apply, and can be calibrated to correct for measurement error. We explore the association between measurements obtained from accurate instruments and those obtained from noisy instruments which can be administered to large groups. We are interested in formulating a model for energy balance by using energy expenditure (EE) and changes in energy stores (ΔES) while accounting for dependence between the two and measurement error. Widely accepted gold standard measurements exist for both EE (DLW) [12, 15, 16, 18–21] and ΔES (DXA) [12, 16, 20]. Table 1 lists abbreviations used in this article. Unfortunately, these instruments are expensive and burdensome. There are alternative approaches [17] to quantify both EE and ΔES that while less expensive and easier to administer, are subject to bias and other errors. Our goal is to model energy balance by using both gold standard and less precise instruments with the end goal of evaluating the error present in the measurements and ultimately calibrating the less precise instruments, so in future studies, researchers can calibrate their measurements of EE and/or ΔES if they are not using a gold standard.

**Table 1:** List of commonly used abbreviations.

EI	Energy Intake
EE	Energy Expenditure
ΔES	Changes in Energy Stores
FM	Fat Mass
FFM	Fat Free Mass
DXA	Dual-energy X-ray absorptiometry
DLW	Doubly Labeled Water

Measurement error modeling is a well developed field in statistics. Fuller made popular linear measurement error models through his book that was the first expose on measurement error [22]. Nonlinear models have since become more popular and widely used and an overview of these models is given in [23]. Berry et al. [24] proposed Bayesian measurement error models that used p-splines to model the relationship between the latent variable and noisy measurements. This was one of the first Bayesian approaches to a problem like this as it was at the onset of the Markov Chain Monte Carlo revolution that allowed for Bayesian modeling to be practical. These models were then extended by [25] and [26] by allowing for a more flexible distribution of the latent variables than a Gaussian as well as using b-splines instead of p-splines. They used Dirichlet Process Mixture Models to allow for more flexibility in the structure of the latent variables, and though simulation and real data anaylsis showed it could have a major effect if the true underlying distribution was not Gaussian. Additionally, they allowed for non-constant variances in the error terms for noisy measurements and gold standard measurements. There is a large body of measurement error research applied to the field of nutrition. Nusser et al. [27] developed a semiparametric approach to estimating intake distributions using noisy, 24 hour recalls of nutrient intakes. Sinha et al. [28] developed Bayesian methods for the analysis of nutritional data that used b-splines and Dirichlet Process Mixtures to allow for flexibility, that would later be extended by [25] and [26]. The analysis of semicontinous data with measurement error was explored in [29], otherwise known as the “NCI method”, and later extended in [30] and [31]. The strong research in measurement error modeling developed for the field of nutrition can be used as a starting point for measurement error modeling in the physical activity realm. Reversible Jump MCMC was designed as a means of model selection [32]. In the context of b-splines, model selection is determining the number of knots and the locations of the knots. An early and practical approach to regression using splines and Reversible Jump MCMC was given in [33], which introduced the idea of Bayesian free-knot splines. Although the method used Reversible Jump MCMC, it was not a “fully Bayesian” approach as it did not place priors on the spline regression coefficients, rather it used OLS to update regression coefficients during each step of the algorithm. A more fully Bayesian approach was given by [34] which allowed for placing priors on the regression coefficients. For complex regression problems where such things as discontinuities in the curve existed, the method of [34] performed better, but with smooth functions that appear to have continuous second derivatives, the simpler to implement method of [33] performed comparably. In these papers, the explanatory variable for which the locations of the knots are being chosen, was assumed to be fixed and known. In this paper, those values will be treated as latent variables which will add a layer of complexity to the algorithm.

In this article we adopt a Bayesian semi-parametric approach. We make distributional assumptions about error terms, but we try to be flexible when modeling the true relationship between less precise measurements and the truth. We propose using free knot splines to model the relationship between the less precise measurements and the truth and we build a Reversible Jump MCMC algorithm to do so. The remainder of this article is organized as follows: in the Methodology section we describe the data structure and assumptions about their dependencies; we also briefly review two commonly used models and introduce a bivariate, Bayesian semi-parametric model that allows for dependence between EE and ΔES. In the Simulated Data and Simulation Study sections, we describe how we simulate complex data and how we constructed the simulation study to assess the performance of the three models. The Results section summarizes our findings in the simulation study. In the Calibration section, we show how calibration could be performed using the proposed model given new data when no gold standard measurements are available.

## Methodology

In this section, a new way to analyze the relationship between gold standard and less expensive measurements that accounts for dependence between EE and ΔES is presented. First, a more precise definition of ΔES is given as well as a practical way to calculate it in practice. Independence assumptions are listed along with justifications that help simplify the model construction. Two simpler models are described before the proposed method: a naïve model that assumes there is no measurement error in gold standard measurements, and a linear measurement error model that assumes a linear relationship between less expensive measurements and the true, latent values of EE and ΔES. Finally, the proposed new model using free knot splines to model the relationship between less expensive measurements and the true, latent values of EE and ΔES is described in further detail.

### Calculation of ΔES

In the energy balance equation,

$$\begin{array}{c} \Delta ES=EI-EE, \end{array}$$ (1)

ΔES is expressed in kcals, and can be positive or negative. To convert DXA measurements of fat mass and fat free mass to kcals, we use Eq (2). Because we assume that energy stores are characterized only as either fat mass (FM) or fat free mass (FFM), this equation provides an exact answer if we know the values of *C*_*FM*_ and *C*_*FFM*_. We let *C*_*FM*_ = 9500 and *C*_*FFM*_ = 1100 like in [20], recognizing that a single value does not account for biological variation. We divide these by the change in time (14 days ± 3 days) and multiply by *C*_*FM*_ and *C*_*FFM*_ to get ΔES in kcals. For each individual, we compute

$$\begin{array}{cc} \Delta ES & =C_{FM}\frac{\Delta FM}{\Delta T}+C_{FFM}\frac{\Delta FFM}{\Delta T}. \end{array}$$ (2)

### Notation

We denote observed average daily EE measured via DLW for subject *i* over time period *j* by $W_{ij}^{EE}$, and observed average daily ΔES measured via DXA for subject *i* over time period *j* by $W_{ij}^{\Delta ES}$. A positive value for ΔES indicates that more calories were taken in than expended. We compute daily values of EE for a person by averaging the total EE for that person obtained by DLW, because DLW gives an estimate of EE over a period of time, in this instance approximately 14 days.

When collecting data on a large population, it is feasible to administer less expensive instruments on most of the subjects. However, they result in less accurate measurements. Although there are several less precise ways to measure EE and ΔES, we keep the notation general since in any given situation we will refer only to one specific instrument. We denote the observed average daily EE obtained with an less precise instrument for subject *i* over time period *j*, $Y_{ij}^{EE}$, and the observed average daily change in energy stores measured by an less precise instrument for subject *i* over time period *j*, $Y_{ij}^{\Delta ES}$.

Lastly, the values which we cannot observe are the *usual* EE and ΔES for subject *i*. We define *usual* as a long run average (expected value) of the true EE and ΔES. Let $X_{i}^{EE}$ represent the *usual* daily EE for subject i and $X_{i}^{\Delta ES}$ represent the *usual* daily ΔES for subject *i*. Note that even if we could observe daily EE and daily Δ*ES* for each participant with no error, there is still within-person variability in these two variables because people change their caloric intake and their physical activity from day to day.

### (In)Dependence assumptions

The observed data vector for subject *i* at time *j* is ($W_{ij}^{EE}$, $W_{ij}^{\Delta ES}$, $Y_{ij}^{EE}$, $Y_{ij}^{\Delta ES}$, *z_i_*) where *Z*_*i*_ is a vector of covariates measured with no error for subject *i*. We start by assuming independence between individuals.

Several of the variables in the model are conditionally independent. Given the value $X_{i}^{EE}$ (*usual* daily EE for subject *i*) and $X_{i}^{\Delta ES}$ (*usual* daily ΔES for subject *i*), and covariates *Z*_*i*_, we assume that:

1. $Y_{ij}^{EE}$ and $Y_{ij}^{\Delta ES}$ are independent of each other,
2. $Y_{ij}^{EE}$ and $Y_{ij}^{\Delta ES}$ are each independent of both $W_{ij}^{EE}$ and $W_{ij}^{\Delta ES}$,
3. $W_{ij}^{EE}$ and $W_{ij}^{\Delta ES}$ are independent of each other.

Assumption 1. follows because given the true values *X* and covariates *Z*, knowing an less precise measurement will give us no more information about the less precise measurement of the other, so long as it is not self-administered. To justify assumption 2., we note that once we know the truth *X*, having an unbiased measurement of *X* will not provide any more information about the less precise, biased measurement of *X*. Assumption 3. follows from a reasoning similar to 1.

### Naïve model

The first model we consider is what we call the naïve model. This model assumes no measurement error in the gold standard instrument, thus DLW and DXA give error-free measurements of $X_{i}^{EE}$ and $X_{i}^{\Delta ES}$, respectively. We also assume that the less precise measurements *Y* are linearly related to the *usual* values and to error free covariates. Based on empirical evidence, gender, BMI, and age all had some effect on the less precise measurement of EE. The naïve model is:

$$(Y_{ij}^{EE}|W_{ij}^{EE},Z_{i},\theta_{yee})\overset{ind}{∼}N\left(\beta_{0,ee}+\beta_{1,ee}W_{ij}^{EE}+\gamma_{ee}Z_{i},\sigma_{\epsilon^{EE}}^{2}\right)$$ (3)

$$(Y_{ij}^{\Delta ES}|W_{ij}^{\Delta ES},Z_{i},\theta_{yes})\overset{ind}{∼}N\left(\beta_{0,es}+\beta_{1,es}W_{ij}^{\Delta ES}+\gamma_{es}Z_{i},\sigma_{\epsilon^{\Delta ES}}^{2}\right).$$ (4)

where the *β*_1,⋅_ terms represents the relationship between less precise measurements and the *usual* EE and ΔES and the *β*_0_ terms represent systematic biases. We let *γ*_⋅_ = (*γ*_1,⋅_, *γ*_2,⋅_, *γ*_3,⋅_) and *γ*_1,⋅_ is the coefficient for gender, *γ*_2,⋅_ is the coefficient for BMI, and *γ*_3,⋅_ is the coefficient for age. We take the standard approach and assume that the errors are normally distributed.

We choose independent priors for all model parameters for all models going forward. Where appropriate, we select priors that are conjugate or conditionally conjugate for ease of implementation but also to permit incorporating weak information through the prior. Prior distributions for all models are listed in the S4 Appendix.

### Linear measurement error model (LMEM)

The Linear Measurement Error Model (LMEM) recognizes that *W*^*EE*^ and *W*^Δ*ES*^ are contaminated with additive measurement error, and are unbiased measurements of truth, rather than equal to truth. Therefore the model becomes hierarchical as it does not directly model the relationship between *Y* and *W*, but rather *Y* and *X* under the assumption that *W* is an unbiased measurement for *X*. The relationship between *Y* and *X* is assumed to be linear, and as in the naïve model, the model also accounts linearly for error-free covariates *Z*. We assume that the measurement errors are normally distributed. To allow dependence between EE and ΔES, we model $\left(X_{i}^{EE},X_{i}^{\Delta ES}\right)$ with a bivariate normal distribution. More formally, the model is given by:

$$\begin{array}{cc} \left(Y_{ij}^{EE}|X_{i}^{EE},Z_{i},\theta_{yee}\right) & \overset{ind}{∼}N\left(\beta_{0,ee}+\beta_{1,ee}X_{i}^{EE}+\gamma_{ee}Z_{i},\sigma_{\epsilon_{EE}}^{2}\right) \end{array}$$ (5)

$$\left(Y_{ij}^{\Delta ES}|X_{i}^{\Delta ES},Z_{i},\theta_{yes})\overset{ind}{\sim }N(\beta_{0,es}+\beta_{1,es}X_{i}^{\Delta ES}+\gamma_{es}Z_{i},\sigma_{\epsilon_{\Delta ES}}^{2}\right)$$ (6)

$$(W_{ij}^{EE}|X_{i}^{EE},Z_{i},\theta_{wee})\overset{ind}{∼}N\left(X_{i}^{EE},\sigma_{\nu_{EE}}^{2}\right)$$ (7)

$$(W_{ij}^{\Delta ES}|X_{i}^{\Delta ES},Z_{i},\theta_{wes})\overset{ind}{∼}N\left(X_{i}^{\Delta ES},\sigma_{\nu_{\Delta ES}}^{2}\right)$$ (8)

$$(X_{i}^{EE},X_{i}^{\Delta ES}|\theta_{X})\overset{ind}{∼}N([\begin{array}{c} \mu_{EE}\\ \mu_{\Delta ES} \end{array}],\Sigma_{X}).$$ (9)

The full likelihood for this model and the one in the next section are givein in the S3 Appendix.

### Spline measurement error model (SMEM)

We extend the LMEM for EE and ΔES in the previous section to include both non-linear and non-parametric components. We follow the same construction of the LMEM to model the gold standard measurements as unbiased for *usual* attributes and subject to normally distributed measurement errors as in (7) and (8).

We wish to understand both the biases as functions of *usual* value and demographic covariates, as well as the measurement error in the instruments themselves. We propose modeling the less precise measurements in a semi-parametric regression framework. Specifically, model the functions *m*_⋅_(⋅) using free knot cubic B-splines, and model demographic covariates with a linear component. We require monotone functions so we can take inverses for calibration later, but this only requires the spline coefficients to be non-decreasing ie. *β*_1_ ≤ *β*_2_ ≤ … ≤ *β*_*k*_ [35] as used in similar applications [28, 36, 37]. Our approach has three benefits. First, the spline is flexible and can pick up an unknown relationship between *X*⋅ and the less precise measurement of the same, which is important because we never observe the truth and therefore it is difficult to justify a particular functional form of the relationship. Second, the use of free knot splines eliminates the need for us to specify the number and position of the knots. Previous methods using splines in measurement error models choose a “moderately large” number of knots, typically at least 15 [24, 26, 28]. We use Reversible Jump MCMC (RJMCMC) to determine the number and position of knots. This means that we treat the number of knots in each regression equation and their knot locations as random variables. Third, the linear component for the covariates allows for an easy interpretation of the parameters and thus the biases in the instrument. We make a working assumption of constant variance for all measurement errors. Based on the above, the model specification is then:

$$(Y_{ij}^{EE}|X_{i}^{EE},Z_{i},\theta_{yee})\overset{ind}{∼}N\left(s_{ee}\left(X_{i}^{EE};\beta_{ee}\right)+\gamma_{ee}Z_{i},\sigma_{\epsilon^{EE}}^{2}\right)$$ (10)

$$(Y_{ij}^{\Delta ES}|X_{i}^{\Delta ES},Z_{i},\theta_{yes})\overset{ind}{∼}N\left(s_{es}\left(X_{i}^{\Delta ES};\beta_{\Delta es}\right)+\gamma_{es}Z_{i},\sigma_{\epsilon^{\Delta ES}}^{2}\right)$$ (11)

$$s_{ee}\left(X_{i}^{EE};\beta_{ee}\right)=\sum_{i=1}^{k_{ee}+4}b_{i,ee}\left(X^{\text{EE}}\right)\beta_{i,ee}=B_{ee}\left(X^{\text{EE}}\right)\beta_{ee}$$ (12)

$$s_{es}\left(X_{i}^{\Delta ES};\beta_{\Delta es}\right)=\sum_{i=1}^{k_{es}+4}b_{i,es}\left(X^{\Delta \text{ES}}\right)\beta_{i,es}=B_{es}\left(X^{\Delta \text{ES}}\right)\beta_{es},$$ (13)

where *B*_*ee*_() and *B*_*es*_() are *n* × (*k*_*ee*_ + 4) and *n* × (*k*_*es*_ + 4) B-spline basis matrices that can be constructed using the recursion specified in [38]. We let *k*_*ee*_ and *k*_*es*_ denote the number of knots for the EE and ΔES splines, respectively.

There are many different types of splines, but we picked B-splines because in similar problems [25, 26, 28] it has been shown that they are numerically more stable than P-splines, for example, which can have major effects on outcomes as compared in [25].

We allow more flexibility in the distribution of the latent variables $(X_{i}^{EE}$, $X_{i}^{\Delta ES})$ by specifying a Dirichlet process mixture prior for them. This allows the data to “speak for themselves” which is ideal when the model includes latent variables. The density of $(X_{i}^{EE}$, $X_{i}^{\Delta ES})$ can then be modeled as an infinite mixture of normals:

$$\left(X_{i}^{EE},X_{i}^{\Delta ES}\right)|\zeta_{i}=h\overset{iid}{∼}N([\begin{array}{c} \mu_{ee,h}\\ \mu_{es,h} \end{array}],\Sigma_{h})$$ (14)

$$\zeta_{i}\overset{iid}{∼}Cat\left(H,\pi \right)$$ (15)

$$V_{h}∼\text{Beta}(1,\alpha )$$ (16)

$$V_{H}=1$$ (17)

$$\pi_{h}=V_{h}\prod_{\ell <h}\left(1-V_{\ell }\right),$$ (18)

where *α* helps control how many components of the infinite mixture are used. We choose to set *α* to 1. The parameter *ζ*_*i*_ takes value for which group observation *i* came. *Cat*(*H*, *π*) is a categorical random variable such that *P*(*ζ*_*i*_ = *h*) = *π*_*h*_, *h* ≤ *H*. In any given problem, we can select *H* such that $\sum_{h=1}^{H}\pi_{h}<\epsilon$ for some *ϵ* > 0 [39], pg. 552.

Although we do not know the true form of the association between the noisy measurements and the usual values, we do not anticipate it to be highly complex, so we would like to use as few knots as necessary. We use *r*_*ee*_ and *r*_*es*_ to denote the knot locations. Our discrete uniform prior on these, means that knots can only occur at the latent values of ($X_{i}^{EE}$, $X_{i}^{\Delta ES}$). This was done largely for computational convenience; we could have assigned a continuous prior for the knot locations, but we do not believe this will adversely affect estimation because the latent ($X_{i}^{EE}$, $X_{i}^{\Delta ES}$) are updated every MCMC iteration. Notice that we have not placed priors on the spline regression coefficients ***β*_*ee*_** and ***β*_*es*_**, or the linear regression coefficients ***γ*_*ee*_** and ***γ*_*es*_**; this is because we will update them using ordinary least squares (OLS). More details can be found in the S2 Appendix.

## Simulated data

In this section we describe how we simulate data to mimic “real” observations, in order to perform a simulation study. Our simulated data need to be sufficiently complex and incorporate dependence in order to faithfully represent the distributions of true EE and EI, as well as gold standard measurements and less precise measurements. We need to simulate data for all the components in the model as well as the latent variables ($X_{i}^{EE}$, $X_{i}^{\Delta ES}$). We explore estimation with measurement errors for the gold standard and less precise measurements under three different scenarios: normal errors, skewed errors, bimodal errors.

For this simulation, we used three covariates: gender, age, BMI. Using a total sample size of 300, we sampled 300 Bernoulli(0.5) to determine gender. Age was simulated from Uniform(20,40). The BMI for an individual was simulated from a Normal(27,5). Let *Z* be the matrix of dimension 300 × 3 that links covariates to individuals.

We simulate ($X_{i}^{EE}$, $X_{i}^{EI}$) from a mixture of 5 bivariate t-distributions. Sixty observation pairs are simulated from five different bivariate t-distributions. The mean and standard deviation of the two-dimensional vector for each of the five t-distributions are each different. The scale matrix for each of the five t-distributions is constant and the degrees of freedom is equal to five.

**Fig 1:**
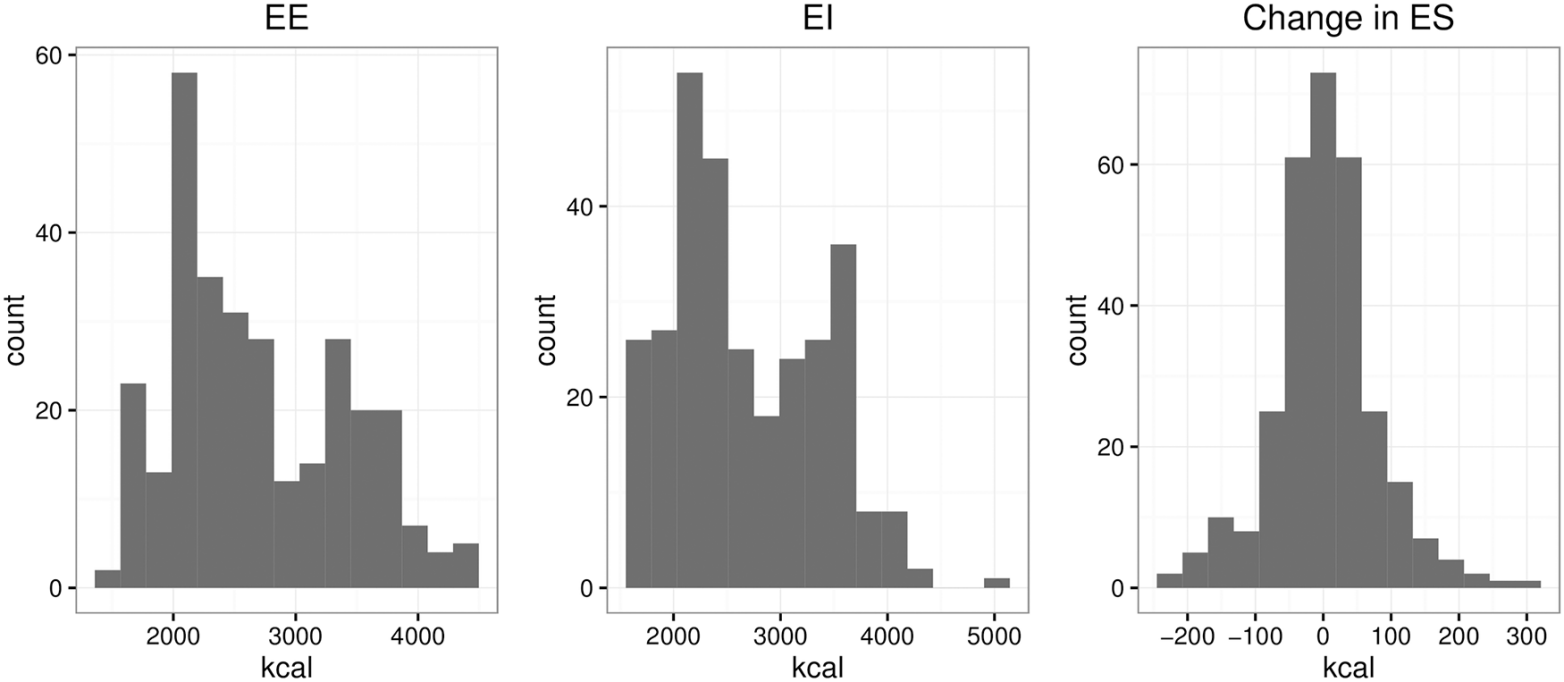
Simulated data distribution. Distribution of simulated latent variables X from one simulated data set.

We let the correlation between EE and EI be 0.4376 as calculated from previous studies’ data. The values used for the vector *γ*_*ee*_ = (300, 14, −7) and *γ*_*es*_ = (−200, 8, −5) for gender, BMI and age, respectively. We compute $X_{i}^{\Delta ES}$ using the energy balance equation in (1). Fig 1 shows histograms f or the latent variables in one simulated data set.

For the gold standard measurements, let

$$\begin{array}{c} \begin{array}{cc} \nu_{ij}^{EE} & =u_{ij}^{EE}+\delta_{ij}^{EE}\\ \nu_{ij}^{\Delta ES} & =u_{ij}^{\Delta ES}+\delta_{ij}^{\Delta ES}, \end{array} \end{array}$$ (19)

where *u*^*EE*^ represents the measurement error in DLW and *u*^Δ*ES*^ represents the measurement error in DXA. Above, $\delta_{ij}^{EE}$ represents the within person deviation in EE for person *i* during time period *j* from the person’s true mean, and $\delta_{ij}^{\Delta ES}$ similarly represents the within person deviation in ΔES for person *i* during time period *j* from the person’s true mean. For the less precise measurements there is a slightly different setup. The within person variability gets added to each individuals’ *usual* values of EE and ΔES and thus is affected by the functions *m*_⋅_(⋅). Therefore we add these within person variation terms *δ* to the *usual* *X* values we simulated to get:

$$\begin{array}{c} \begin{array}{cc} X_{ij}^{EE} & =X_{i}^{EE}+\delta_{ij}^{EE}\\ X_{ij}^{\Delta ES} & =X_{i}^{\Delta ES}+\delta_{ij}^{\Delta ES}, \end{array} \end{array}$$ (20)

and the functions *m*_⋅_(⋅) depend on $X_{ij}^{\cdot }$.

The pairs ($\delta_{ij}^{EE}$, $\delta_{ij}^{\Delta ES}$) are simulated jointly but independently across time and individual. We simulate the within person variability terms ($\delta_{ij}^{EE}$, $\delta_{ij}^{\Delta ES}$) from a bivariate normal distribution.

We assume that DLW and DXA are unbiased measurements of EE and ΔES, respectively. These measurements are simulated according to (7) and (8) where we further brake down *ν* as in (19). The *u* term represents the measurement error components we still need to specify and *δ* represents the within person component of the error which we have already discussed. We assume that the *u* terms are independent within and across individuals as well as of all *δ* and *X*.

From these simulated values, we then get simulated gold standard data $W_{ij}^{EE}$, $W_{ij}^{\Delta ES}$. We generate measurement errors for the gold standard measurements (and for the less precise measurements) from three different distributions: normal, skewed normal, and a bimodal mixture of two normals that is centered around 0. Parameters were chosen such that the means of all error distributions are 0, and the variances for each distribution is the same within EE errors and within ΔES errors.

We generate observations for less precise measurements in a similar fashion as in the last section. We assume that the errors are independent within and across subjects as well as mutually independent with all *δ*, *X* and *Z* terms. We draw these errors from densities that are the same to those in the previous section, except with larger variances.

In contrast to the gold standard measurements which we assume are unbiased, we now add bias to the less precise measurements. The bias is introduced via the functions *m*_*ee*_ and *m*_*es*_. For these simulated data, we let:

$$\begin{array}{c} m_{ee}\left(X,Z\right)=2X_{i}^{EE}-\frac{4000}{1+e^{-0.002X_{i}^{EE}-2200}}, \end{array}$$ (21)

$$\begin{array}{c} m_{es}\left(X,Z\right)=\frac{1000}{1+e^{-0.04X_{i}^{\Delta ES}}}-2000+X_{i}^{\Delta ES}. \end{array}$$ (22)

**Fig 2:**
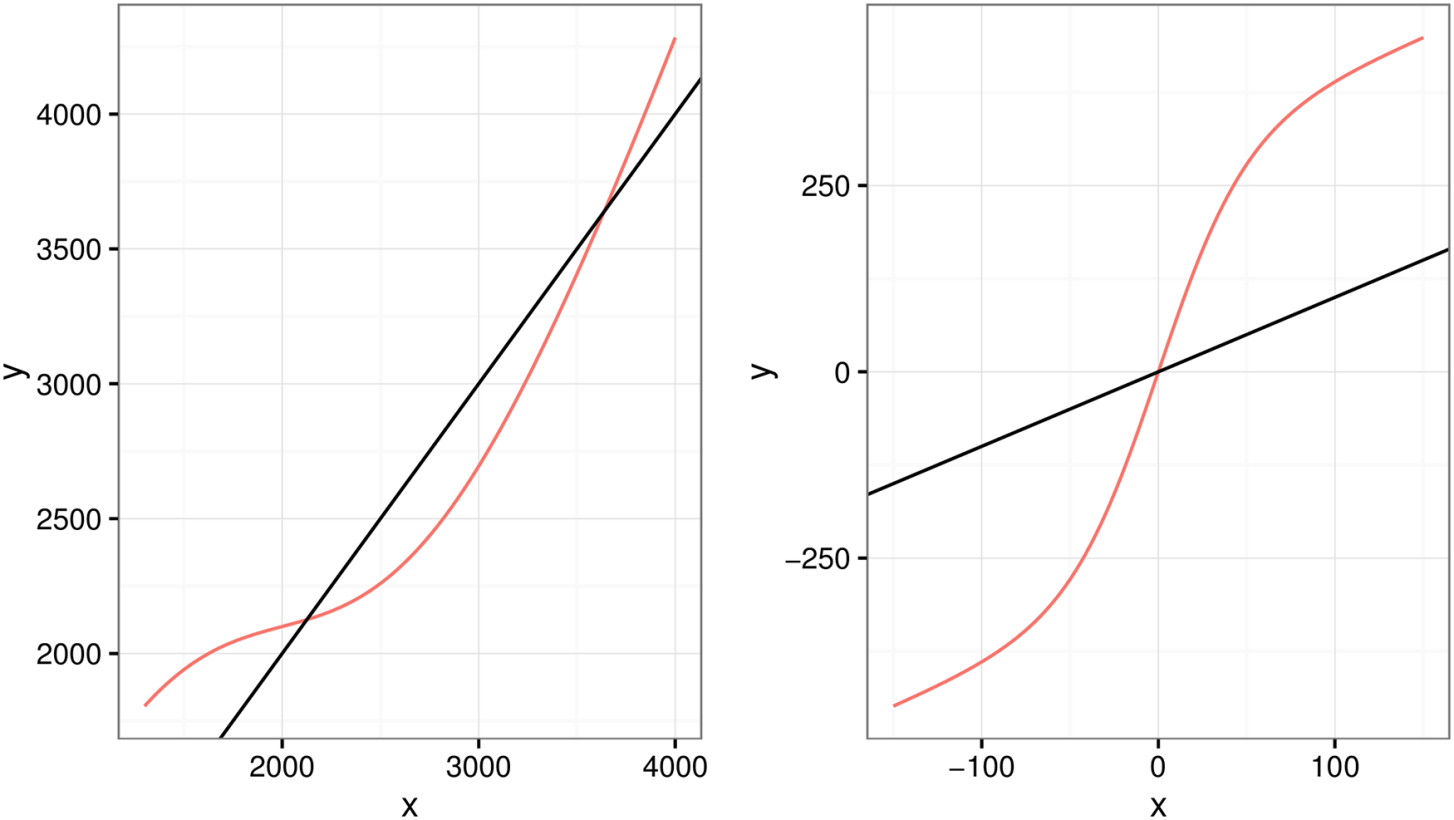
Nonlinear functions. Plot of nonlinear functions *m*_*ee*_() (left) and *m*_*es*_() (right), and Y = X is black for reference to unbiased measurement.

Fig 2 shows *m*_*ee*_(⋅) on the left and *m*_*es*_(⋅) on the right both against a *y* = *x* line for comparison.

We then add *Z*_*i*_ *γ*_⋅_ to the simulated less precise measures of EE and ΔES.

## Estimation

We adopt a Bayesian approach to estimation in this problem, and therefore, our goal is to estimate the joint posterior distribution of all parameters and latent variables in the model. In our case, the joint posterior distribution is *p*(***θ***, *X*^*EE*^, *X*^Δ*ES*^|*W*^*EE*^, *W*^Δ*ES*^, *Y*^*EE*^, *Y*^Δ*ES*^, *Z*). We use Markov Chain Monte Carlo (MCMC) methods to approximate the posterior distribution. For the naïve and LMEM models, we used Just Another Gibbs Sampler (JAGS) to simulate draws from the posterior distribution. This was simple to implement and was relatively quick to sample. In order to fit free knot splines which allow for dimension change, we must use Reversible Jump MCMC which requires a more complex sampler. We use R and C++ for the RJMCMC sampler. Because the algorithms are technical and not the main objectives of this paper, we provide the algorithm for the Gibbs sampler in the S1 Appendix and the reversible jump algorithm in the S2 Appendix.

## Simulation study

In this section we describe a simulation study that we carried out, to check the performance of the models we propose. We are interested in the predictive performance of the models because our main goal is to develop a calibration tool. We are also interested in evaluating the robustness of the model to departures of the errors from the standard normality assumption, which is why we simulate errors from two alternative error distributions. We present performance measures such as predicted mean squared error (PMSE) for the regression function in question as well as posterior means and posterior standard deviations for parameters of interest.

### Setup

We simulated 200 data sets each for normal, skewed, and bimodal errors for both 2 and 4 replicate measurements per individual. The number of individuals is 300 in all cases. Preliminary analysis suggest that the number of replicates per individual has a stronger impact on performance than the number of individuals.

Although we would like to be as flexible as possible with our distributional assumptions on the bivariate latent variables, we also want a model that produces estimates with low prediction mean squared error (PMSE) given the data constraints of our application. In practice, it is difficult to obtain more than two replicate measurements on an individual, at least when using the gold standard measurements. During the simulation study, we found that the Dirichlet Process prior on the latent variables produced unstable results in parameter estimates and low acceptance rates of proposals in the random walk Metropolis-Hastings algorithm when we only had two replicate observations per person. Results were stable however, when four replicates per person were available. Because of this issue, we fit a fourth model using a bivariate normal distribution for the prior of latent variables instead of the Dirichlet Process prior while still using splines for the regression functions. We refer to this model as SMEMN. The MCMC has a minor change in the Gibbs step (steps (a)-(c) are eliminated and step (d) no longer depends on grouping *h*).

We set the values of the hyperparameters as follows: $M_{\beta_{0,ee}}=M_{\beta_{0,es}}=0$, $C_{\beta_{0,ee}}=C_{\beta_{0,es}}=100000$, $M_{\beta_{1,ee}}=M_{\beta_{1,es}}=1$, $C_{\beta_{1,ee}}=C_{\beta_{1,es}}=100000$, $M_{\gamma_{ee}}=M_{\gamma_{es}}=0$, $C_{\gamma_{ee}}=C_{\gamma_{es}}=100000$, *a_yee_* = *a_yes_* = *a_wee_* = *a_wes_* = *b_yee_* = *b_yes_* = *b_wee_* = *b_wes_* = 0.1, *ψ* = *I*_2×2_, *d* = 3, *M_μ_* = (2400, 0), *C_μ_* = *diag*(100000, 100000), λ_*ee*_ = λ_*es*_ = 1. We ran the MCMC for 3 chains of 12,000 iterations, using the first 2000 as burn in, and convergence for all models was fast as indicated by trace plots and Gelman-Rubin diagnostics less than 1.04.

### Results

Tables 2, 3 and 4 show results averaged over 200 Monte Carlo samples, for normal, skewed, and bimodal errors, respectively. The asterisk next to the truth for the measurement error with respect to the less precise measurements indicates that this is a Monte Carlo approximation to the truth. Recall that we included within person variation in the functions *m*_⋅_(⋅), but in our model we use the working assumption that the additive error term accounts for both within person variability and measurement error. Because we cannot directly extract the value from the function, we approximate it by generating 10,000 data sets and removing the mean function from the less precise observations, and then calculating the standard deviation of the residual. We then averaged those standard deviation estimates to get the one reported in the table.

**Table 2:** Summary of simulation under normal errors for naïve, LMEM, SMEMN, SMEM models, respectively.

**Naïve**	*σ*_*yee*_		*σ*_*yes*_						*γ*_1,*ee*_		*γ*_2,*ee*_		*γ*_3,*ee*_		*γ*_1,*es*_		*γ*_2,*es*_		*γ*_3,*es*_	
Replicates	2	4	2	4					2	4	2	4	2	4	2	4	2	4	2	4
Mean Est	477.65	473.42	347.49	354.85					254.67	248.66	14.88	14.03	-4.14	-5.29	-200.53	-199.39	7.91	8.31	-4.94	-5.13
Std Err	17.82	19.24	9.43	6.80					43.65	36.10	4.33	3.62	3.37	3.06	28.19	22.83	2.90	2.13	2.35	1.73
Bias	72.15	67.92	13.49	20.85					-45.33	-51.34	0.88	0.03	2.86	1.71	-0.53	0.61	-0.09	0.31	0.06	-0.13
Truth	405.50	405.50	334.00	334.00					300.00	300.00	14.00	14.00	-7.00	-7.00	-200.00	-200.00	8.00	8.00	-5.00	-5.00
**LMEM**	*σ*_*yee*_		*σ*_*yes*_		*σ*_*wee*_		*σ*_*wes*_		*γ*_1,*ee*_		*γ*_2,*ee*_		*γ*_3,*ee*_		*γ*_1,*es*_		*γ*_2,*es*_		*γ*_3,*es*_	
Replicates	2	4	2	4	2	4	2	4	2	4	2	4	2	4	2	4	2	4	2	4
Mean Est	444.34	446.63	320.41	338.26	255.70	255.85	69.18	71.81	249.50	240.63	14.30	13.67	-4.50	-5.28	-199.27	-198.49	7.91	8.29	-4.91	-5.15
Std Err	16.84	14.22	10.76	7.53	10.74	6.33	2.27	1.56	43.44	37.02	4.25	3.60	3.38	3.04	28.31	22.96	2.91	2.12	2.30	1.71
Bias	38.84	41.13	-13.59	4.26	5.70	5.85	-3.68	-1.05	-50.50	-59.37	0.30	-0.33	2.50	1.72	0.73	1.51	-0.09	0.29	0.09	-0.15
Truth	405.50	405.50	334.00	334.00	250.00	250.00	72.86	72.86	300.00	300.00	14.00	14.00	-7.00	-7.00	-200.00	-200.00	8.00	8.00	-5.00	-5.00
**SMEMN**	*σ*_*yee*_		*σ*_*yes*_		*σ*_*wee*_		*σ*_*wes*_		*γ*_1,*ee*_		*γ*_2,*ee*_		*γ*_3,*ee*_		*γ*_1,*es*_		*γ*_2,*es*_		*γ*_3,*es*_	
Replicates	2	4	2	4	2	4	2	4	2	4	2	4	2	4	2	4	2	4	2	4
Mean Est	393.69	400.55	313.47	331.79	246.81	248.93	67.61	71.04	293.16	294.61	14.17	14.11	-6.86	-6.78	-200.07	-200.86	8.00	8.04	-4.95	-5.26
Std Err	11.52	8.29	12.00	8.27	8.78	5.79	2.32	1.66	36.16	26.19	3.50	2.42	2.86	2.16	26.86	19.44	2.49	2.06	2.11	1.45
Bias	-11.81	-4.95	-20.53	-2.21	-3.19	-1.07	-5.25	-1.82	-6.84	-5.39	0.17	0.11	0.14	0.22	-0.07	-0.86	0.00	0.04	0.05	-0.26
Truth	405.50	405.50	334.00	334.00	250.00	250.00	72.86	72.86	300.00	300.00	14.00	14.00	-7.00	-7.00	-200.00	-200.00	8.00	8.00	-5.00	-5.00
**SMEM**		*σ*_*yee*_		*σ*_*yes*_		*σ*_*wee*_		*σ*_*wes*_		*γ*_1,*ee*_		*γ*_2,*ee*_		*γ*_3,*ee*_		*γ*_1,*es*_		*γ*_2,*es*_		*γ*_3,*es*_
Replicates		4		4		4		4		4		4		4		4		4		4
Mean Est		400.13		331.38		248.94		70.85		297.62		14.16		-7.11		-198.04		8.12		-4.94
Std Err		8.25		8.43		6.05		1.62		26.53		2.72		2.08		18.11		1.85		1.64
Bias		-5.37		-2.62		-1.06		-2.01		-2.38		0.16		-0.11		1.96		0.12		0.06
Truth		405.50		334.00		250.00		72.86		300.00		14.00		-7.00		-200.00		8.00		-5.00

**Table 3:** Summary of simulation under skewed errors for naïve, LMEM, SMEMN, SMEM models, respectively.

**Naïve**	*σ*_*yee*_		*σ*_*yes*_						*γ*_1,*ee*_		*γ*_2,*ee*_		*γ*_3,*ee*_		*γ*_1,*es*_		*γ*_2,*es*_		*γ*_3,*es*_	
Replicates	2	4	2	4					2	4	2	4	2	4	2	4	2	4	2	4
Mean Est	473.87	466.80	311.66	317.05					255.46	250.72	14.08	13.23	-5.05	-6.04	-197.98	-200.46	7.79	7.91	-4.98	-4.92
Std Err	17.36	13.06	9.08	6.64					40.99	32.70	3.99	3.53	3.38	3.02	24.90	19.73	2.45	1.78	2.28	1.67
Bias	68.37	61.30	-22.34	-16.95					-44.54	-49.28	0.08	-0.77	1.95	0.96	2.02	-0.46	-0.21	-0.09	0.02	0.08
Truth	405.50	405.50	334.00	334.00					300.00	300.00	14.00	14.00	-7.00	-7.00	-200.00	-200.00	8.00	8.00	-5.00	-5.00
**LMEM**	*σ*_*yee*_		*σ*_*yes*_		*σ*_*wee*_		*σ*_*wes*_		*γ*_1,*ee*_		*γ*_2,*ee*_		*γ*_3,*ee*_		*γ*_1,*es*_		*γ*_2,*es*_		*γ*_3,*es*_	
Replicates	2	4	2	4	2	4	2	4	2	4	2	4	2	4	2	4	2	4	2	4
Mean Est	432.67	435.88	289.34	304.25	254.13	255.00	69.67	71.68	251.72	244.04	13.38	12.88	-5.46	-6.10	-196.76	-200.05	7.82	7.89	-4.96	-4.94
Std Err	16.70	12.10	9.90	7.05	11.99	6.27	2.63	1.80	41.34	33.01	3.95	3.50	3.35	3.00	25.32	19.53	2.39	1.77	2.25	1.67
Bias	27.17	30.38	-44.66	-29.75	4.13	5.00	-3.20	-1.18	-48.28	-55.96	-0.62	-1.12	1.54	0.90	3.24	-0.05	-0.18	-0.11	0.04	0.06
Truth	405.50	405.50	334.00	334.00	250.00	250.00	72.86	72.86	300.00	300.00	14.00	14.00	-7.00	-7.00	-200.00	-200.00	8.00	8.00	-5.00	-5.00
**SMEMN**	*σ*_*yee*_		*σ*_*yes*_		*σ*_*wee*_		*σ*_*wes*_		*γ*_1,*ee*_		*γ*_2,*ee*_		*γ*_3,*ee*_		*γ*_1,*es*_		*γ*_2,*es*_		*γ*_3,*es*_	
Replicates	2	4	2	4	2	4	2	4	2	4	2	4	2	4	2	4	2	4	2	4
Mean Est	393.56	402.91	282.68	298.37	247.00	248.83	68.93	71.23	306.58	305.65	14.27	14.44	-6.95	-7.14	-197.44	-198.85	7.76	8.04	-4.92	-4.86
Std Err	13.33	9.56	10.96	7.47	9.54	6.33	2.56	1.69	36.09	27.06	3.35	2.57	2.77	2.31	24.41	17.81	2.42	1.73	2.10	1.47
Bias	-11.94	-2.59	-51.32	-35.63	-3.00	-1.17	-3.93	-1.63	6.58	5.65	0.27	0.44	0.05	-0.14	2.56	1.15	-0.24	0.04	0.08	0.14
Truth	405.50	405.50	334.00	334.00	250.00	250.00	72.86	72.86	300.00	300.00	14.00	14.00	-7.00	-7.00	-200.00	-200.00	8.00	8.00	-5.00	-5.00
**SMEM**		*σ*_*yee*_		*σ*_*yes*_		*σ*_*wee*_		*σ*_*wes*_		*γ*_1,*ee*_		*γ*_2,*ee*_		*γ*_3,*ee*_		*γ*_1,*es*_		*γ*_2,*es*_		*γ*_3,*es*_
Replicates		4		4		4		4		4		4		4		4		4		4
Mean Est		403.10		298.16		248.70		71.11		313.22		14.13		-7.26		-199.31		8.10		-4.85
Std Err		8.25		7.01		6.67		1.57		27.80		2.37		2.13		17.70		1.54		1.54
Bias		-2.40		-35.84		-1.30		-1.75		13.22		0.13		-0.26		0.69		0.10		0.15
Truth		405.50		334.00		250.00		72.86		300.00		14.00		-7.00		-200.00		8.00		-5.00

**Table 4:** Summary of simulation under bimodal errors for naïve, LMEM, SMEMN, SMEM models, respectively.

**Naïve**	*σ*_*yee*_		*σ*_*yes*_						*γ*_1,*ee*_		*γ*_2,*ee*_		*γ*_3,*ee*_		*γ*_1,*es*_		*γ*_2,*es*_		*γ*_3,*es*_	
Replicates	2	4	2	4					2	4	2	4	2	4	2	4	2	4	2	4
Mean Est	342.63	344.52	233.52	246.69					227.08	221.48	12.15	12.22	-5.61	-5.52	-198.19	-199.20	8.01	8.13	-4.84	-4.99
Std Err	12.61	15.26	6.43	5.24					32.41	29.92	3.28	3.21	3.05	2.94	17.40	15.19	1.64	1.59	1.49	1.23
Bias	-62.87	-60.98	-100.48	-87.31					-72.92	-78.52	-1.85	-1.78	1.39	1.48	1.81	0.80	0.01	0.13	0.16	0.01
Truth	405.50	405.50	334.00	334.00					300.00	300.00	14.00	14.00	-7.00	-7.00	-200.00	-200.00	8.00	8.00	-5.00	-5.00
**LMEM**	*σ*_*yee*_		*σ*_*yes*_		*σ*_*wee*_		*σ*_*wes*_		*γ*_1,*ee*_		*γ*_2,*ee*_		*γ*_3,*ee*_		*γ*_1,*es*_		*γ*_2,*es*_		*γ*_3,*es*_	
Replicates	2	4	2	4	2	4	2	4	2	4	2	4	2	4	2	4	2	4	2	4
Mean Est	264.16	265.06	220.65	237.60	207.41	207.86	52.92	56.11	216.24	203.84	11.50	11.52	-5.75	-5.38	-201.48	-201.44	7.90	8.07	-4.74	-4.92
Std Err	15.07	8.86	7.31	5.56	11.92	8.43	1.85	1.26	32.90	30.36	3.20	3.25	3.07	2.91	17.18	15.06	1.66	1.57	1.49	1.23
Bias	-141.34	-140.44	-113.35	-96.40	-42.59	-42.14	-19.94	-16.75	-83.76	-96.16	-2.50	-2.48	1.25	1.62	-1.48	-1.44	-0.10	0.07	0.26	0.08
Truth	405.50	405.50	334.00	334.00	250.00	250.00	72.86	72.86	300.00	300.00	14.00	14.00	-7.00	-7.00	-200.00	-200.00	8.00	8.00	-5.00	-5.00
**SMEMN**	*σ*_*yee*_		*σ*_*yes*_		*σ*_*wee*_		*σ*_*wes*_		*γ*_1,*ee*_		*γ*_2,*ee*_		*γ*_3,*ee*_		*γ*_1,*es*_		*γ*_2,*es*_		*γ*_3,*es*_	
Replicates	2	4	2	4	2	4	2	4	2	4	2	4	2	4	2	4	2	4	2	4
Mean Est	257.20	256.24	217.62	235.09	182.30	189.38	51.86	55.39	220.14	211.17	12.25	11.96	-5.90	-5.81	-201.34	-200.20	7.87	7.98	-5.08	-4.95
Std Err	11.41	10.47	7.51	5.46	8.22	5.74	1.87	1.19	30.56	32.63	3.24	2.84	2.86	2.47	17.33	13.18	1.60	1.28	1.46	1.13
Bias	-148.30	-149.26	-116.38	-98.91	-67.70	-60.62	-21.01	-17.47	-79.86	-88.83	-1.75	-2.04	1.10	1.19	-1.34	-0.20	-0.13	-0.02	-0.08	0.05
Truth	405.50	405.50	334.00	334.00	250.00	250.00	72.86	72.86	300.00	300.00	14.00	14.00	-7.00	-7.00	-200.00	-200.00	8.00	8.00	-5.00	-5.00
**SMEM**		*σ*_*yee*_		*σ*_*yes*_		*σ*_*wee*_		*σ*_*wes*_		*γ*_1,*ee*_		*γ*_2,*ee*_		*γ*_3,*ee*_		*γ*_1,*es*_		*γ*_2,*es*_		*γ*_3,*es*_
Replicates		4		4		4		4		4		4		4		4		4		4
Mean Est		251.97		235.27		191.80		55.25		218.08		12.43		-5.67		-202.35		8.01		-5.14
Std Err		12.67		5.58		6.59		1.30		37.21		2.84		2.34		12.93		1.27		1.13
Bias		-153.53		-98.73		-58.20		-17.62		-81.92		-1.57		1.33		-2.35		0.01		-0.14
Truth		405.50		334.00		250.00		72.86		300.00		14.00		-7.00		-200.00		8.00		-5.00

Across all models and error types, the linear coefficients are estimated largely without bias. This is not too surprising since these covariates are measured without error. This suggestst the regression coefficient estimates will not be affected by distribution of the errors. Additionally, the regression coefficients can be interpreted as biases inherent to the device. For example, *γ*_1,*ee*_ can be thought of as the the additional number of calories a device will report for a male compared to a female, all else equal. These results could be informative and useful as a secondary study goal. The biases and standard errors are slightly smaller for models SMEMN and SMEM, however. All three measurement error models perform about the same when assessing the measurement error in the gold standard instruments. When errors are generated from a bimodal distribution, estimated error variances are biased toward zero. This is true for the measurement error in the less precise measurements as well. The SMEMN and SMEM models produce similar results for the estimates of variance measurement error of less precise measurements. Estimates are good for EE and ΔES when errors are normal, but biased low for ΔES for both skewed and bimodal errors. Both the naïve model and the linear measurement error model result in estimated measurement error standard deviations for the less precise measurement that are too large under normal errors and skewed errors for EE. When the departure from normality is significant (bimodal error distribution) unbiasedly estimating the measurement error variance can be challenging.

**Fig 3:**
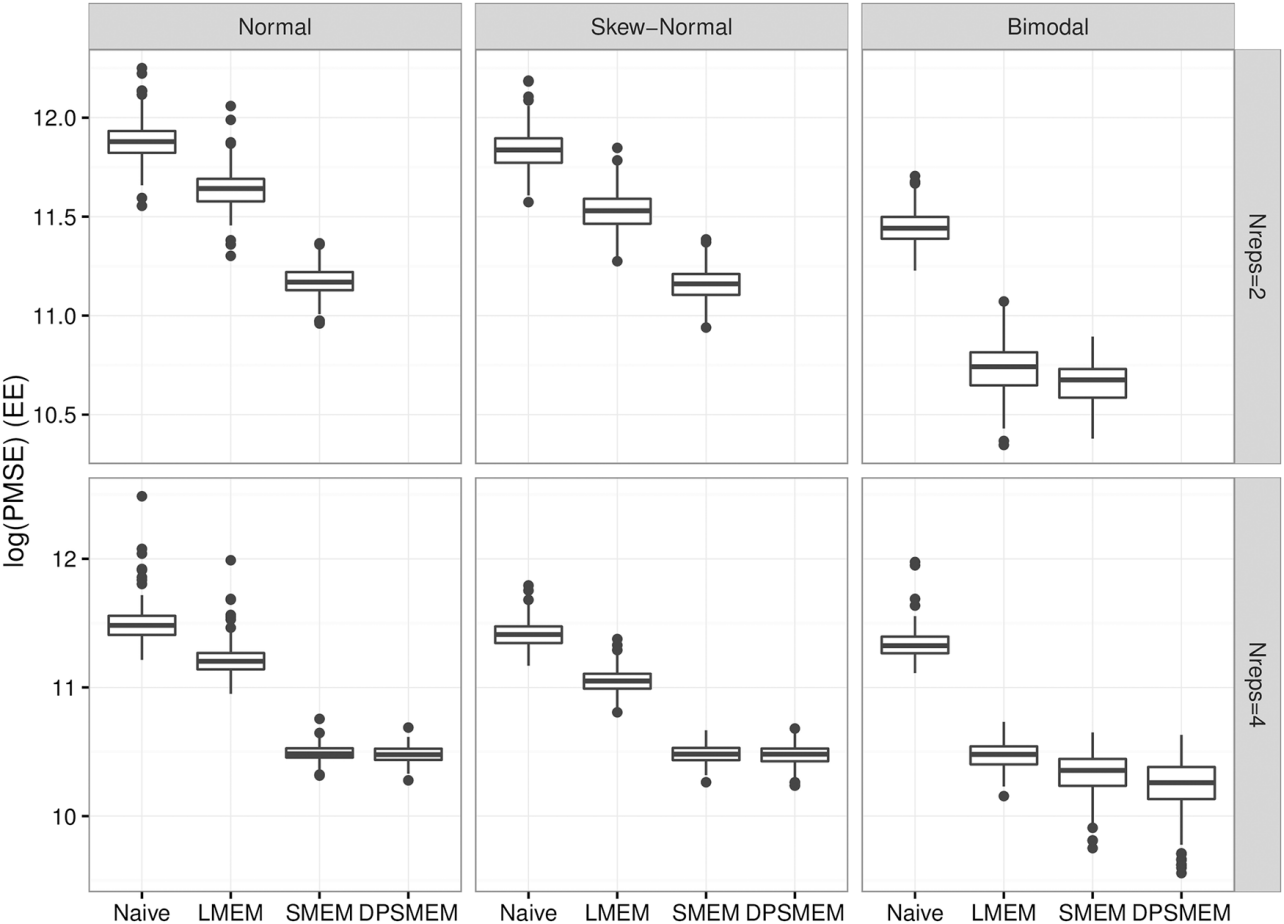
PMSE for EE. Log PMSE for EE Regression faceted by measurement error distribution and number of replicates.

**Fig 4:**
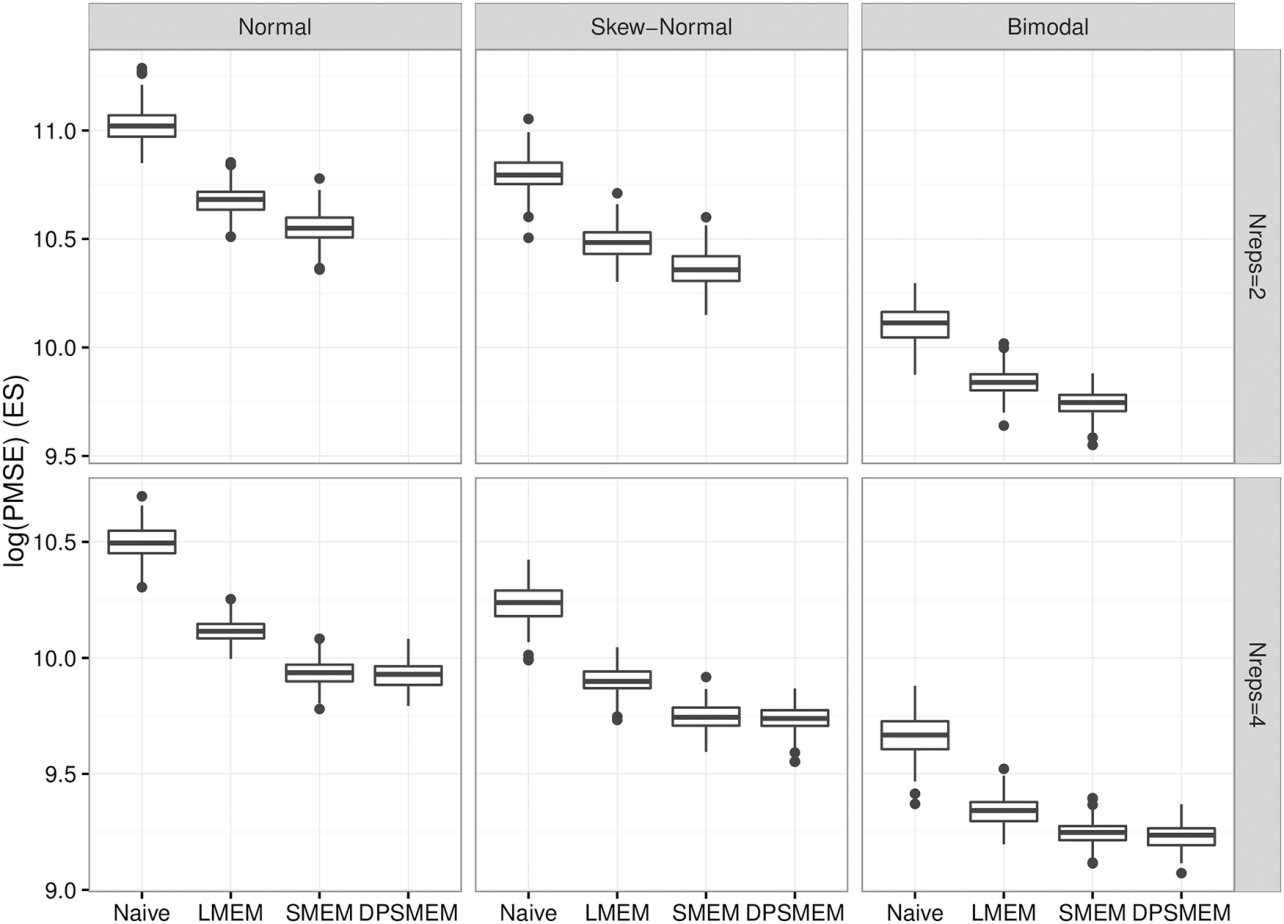
PMSE for ΔES. Log PMSE for ΔES Regression faceted by measurement error distribution and number of replicates.

Fig 3 shows boxplots of the log mean PMSE for each simulation for each model under each type of error distribution for EE for 2 and 4 replicates, and Fig 4 shows the same for ΔES. There is a consistent decreasing pattern from simpler to most complex in terms of the models. First, the naïve model does much worse than the same model which accounts for measurement error. The naïve model and the linear measurement error model perform much worse than the models with free knot splines in terms of PMSE. This is under the case where the true relationship is non-linear, but when looking at the noisy data the relationship doesn’t appear to be highly non-linear. This suggests the methods using free knot splines are able to see potential relationships that are difficult to see with only the noisy data. There is not a large difference between the SMEMN and SMEM model in terms of PMSE, but the SMEM model generally does better. There are more parameters in SMEM to help explain the scientific mechanism of the problem, but that does not necessarily imply better prediction. The question is whether the small improvement is worth the increase in model complexity. We think that the answer is no for two reasons: (i) our main focus with this model is calibrating the less precise measurements and not necessarily conducting inference at the latent variable level, and (ii) the DP approach is reliable only situations when we have four replicates, which for gold standard measurements, is unrealistic in practice. Because the main focus is to calibrate less precise measurements, the simulation results are promising.

**Fig 5:**
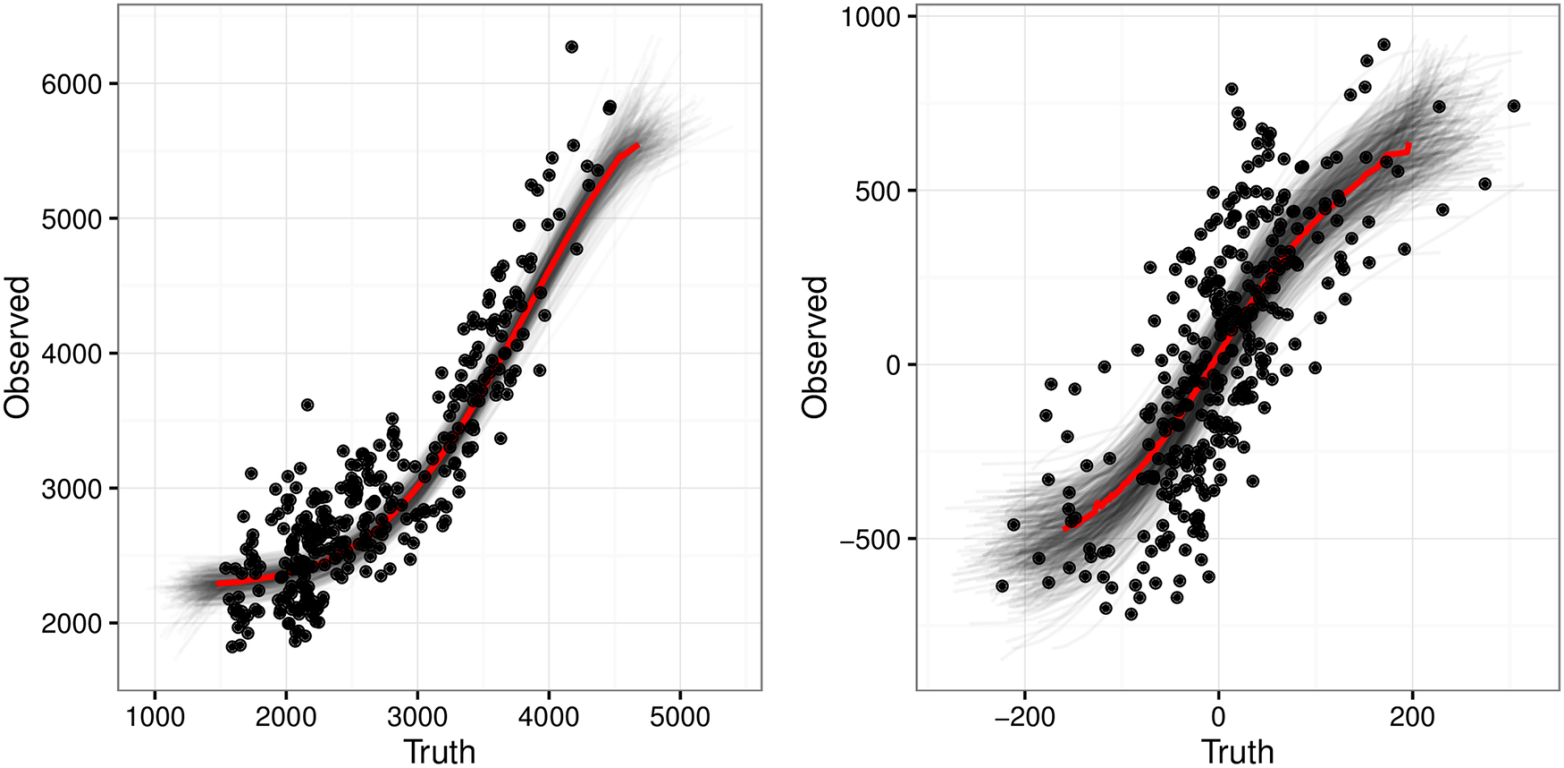
Fitted spline. Spline function for Model SMEMN with Skewed Errors.

**Fig 6:**
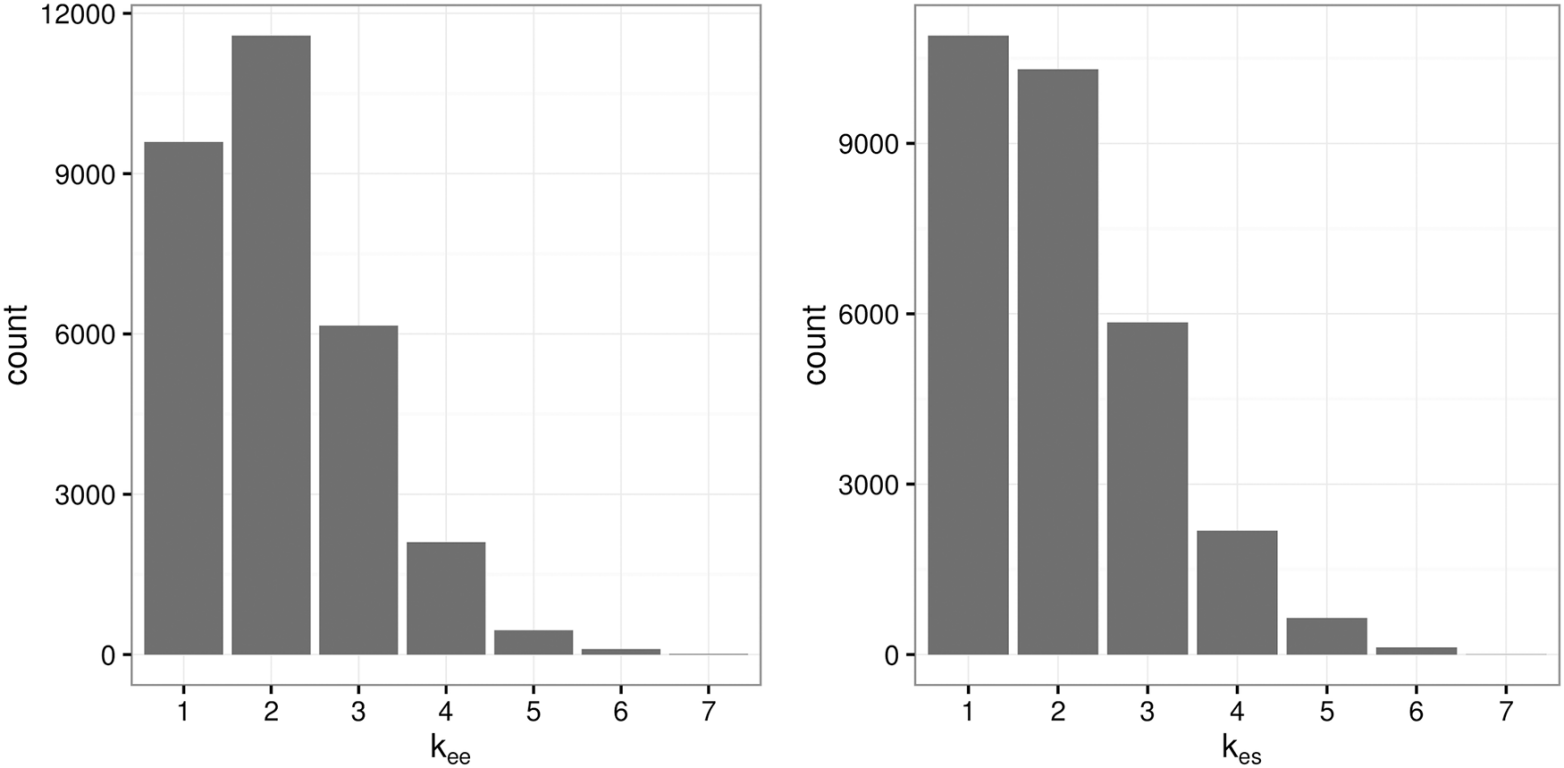
Distribution of *k*_*ee*_ and *k*_*es*_. Distribution of Number of knots for Model SMEMN with Skewed Errors.

To see the structure of the nonlinear model with the fitted spline on top of the simulated data, we provide plots from one of the 200 simulated data sets. We chose a simulated data set with skewed errors and two replicates per person. Fig 5 shows the fitted spline between the values of EE and ΔES and the measurements obtained with the less precise measurement. The points correspond to the individual simulated data where the y value is the mean of the two replicates. The bold (red) line is the mean estimated spline function. We randomly selected 500 MCMC draws for the spline, and plotted them behind the mean. Fig 6 gives the distribution of the number of knots for the spline for both the EE and ΔES splines. The splines are not overly complex and typically use four or fewer knots.

## Calibration

The main goal of this work is to develop a calibration approach to “correct” the measurements of EE ΔES obtained with less precise, noisy measurements. That is, given a measurement of EE or ΔES from an less precise instrument and some demographic information, we can return a better estimate of the true value as well as a credible interval that shows the uncertainty in the estimate. Calibration for our models simply amounts to finding the inverse of the fitted models as a function of *Y* instead of *X*, and *Z*. For a given observed value of *Y* and *Z*, and an estimate of *γ*, the calibration for *X* is:

$$X_{calibrated}=s^{-1}\left(y-\gamma^{\prime }Z\right).$$ (23)

We cannot find the inverse in (23) in closed form so we find it numerically instead. To do so, we use optimize in R for the function |*s*(*x*) − y* | where *s*() represents the regression function and y* is the observed less precise measurement minus the vector of coefficients *γ* multiplied by the individuals’ covariate values *Z*. The algorithm for our calibration for individual *i* is as follows:

For r = 1,…R

1. Calculate $y_{i}^{*}=y_{i}-\gamma^{{\left(r\right)}^{\prime }}Z_{i}$, where *Z*_*i*_ are the covariate values for individual *i*.
2. Use optimize for the function $|s_{i}\left(x\right)-y_{i}^{*}|$ to choose the value of *x* that will minimize the criterion, call this *x*_*i*,*calibrated*_. Here, *s*_*i*_(*x*) is the predicted value of *y*_*i*_ for the given value *x* using the MCMC draw for the spline coefficients ***β***.^(*r*)^, latent variables (*X*^*EE*(*r*)^, *X*^Δ*ES*(*r*)^), and knot locations ($r_{ee}^{\left(r\right)}$, $r_{es}^{\left(r\right)}$) from the *r*^*th*^ draw of the chain.

Since our interest lies in correcting less expensive measurements for potentially non-linear biases and measurement error as determined jointly in the model through the use of gold standard measurements, this calibration step is of most interest to practitioners. Although parameters estimates from the model may be interesting, obesity, nutrition, and physical activity researchers often need reliable data on EI and EE to understand the effects of treatments in controlled experiments or relationships found in exploratory analyses from observational data. The calibration method above along with the estimated posterior distribution for the model gives practitioners a powerful way to adjust their measurements of EI and/or EE for measurement error.

**Fig 7:**
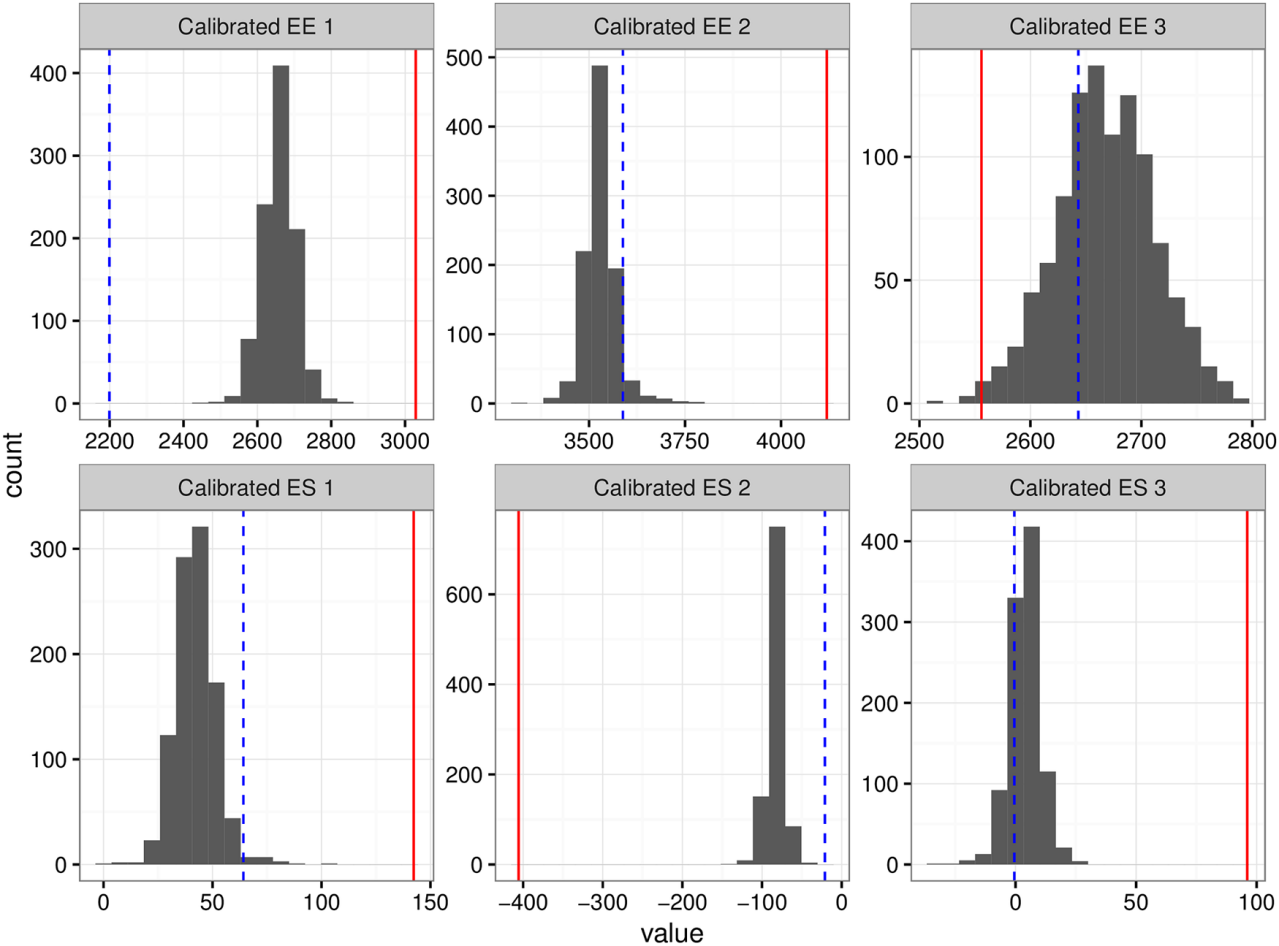
Calibration. Posteriors of calibrated observations. Solid vertical line shows observed value from less precise measurement and dashed vertical line shows truth.

As an example, suppose that we wish to calibrate three noisy measurements each from a different individual using Model SMEMN. We randomly select 3 individuals from the same data set used earlier to give results for model SMEMN. Individual 1 is male, BMI of 28.6, age 20.5; individual 2 is female, BMI of 21.5, age 30.1 and individual 3 is male, BMI 38.6 and age 22.8. Observed less precise measurements for these individuals, their true values, as well as 95% credible intervals for their mean calibrated truth under skewed normal errors are given in Table 5. Fig 7 shows histograms of 1000 calibrated draws for each individual for EE and ΔES measurements under skewed errors. Looking at the table and figure, one can see that the calibration helps pull the less precise measurement closer to the truth. In all cases, the calibration helped to improve the estimate obtained from the less precise measurement. A simple point estimate correction may be used and an analysis could procede with these corrected measurements taken as truth; a more comprehensive approach would be to use the point estimate of EE and ΔES as well as the uncertainty given by the posterior distribution. This would allow for an approach that fully accounts for biases and measurement error uncertainty present in the data as to avoid making erroneous conclusions based on bad data. Running this on many of the simulated individuals had similar results.

**Table 5:** 95% credible interval for calibration estimate for less precise measurements for skewed errors.

Person		Lower	Median	Upper	Observed	True Value
1	EE	2574.18	2666.00	2736.39	3028.89	2199.25
2		3452.51	3525.18	3619.08	4119.26	3588.12
3		2571.99	2665.46	2744.65	2555.86	2643.14
1	ΔES	25.15	42.35	60.57	142.30	64.17
2		-104.21	-82.93	-63.90	-405.74	-21.08
3		-8.41	3.91	17.83	96.06	-0.48

## Discussion

In this chapter we presented a semi-parametric approach to model energy balance via its components EE and ΔES. We assume that we have gold standards for both quantities that are unbiased, as well as less precise instruments that result in biased measurements of the truth. We propose a model where the form of the association between the unbiased and the biased measurements of EE (or of ΔES) is left unspecified and uses splines to estimate that function. This allows a flexible relationship between an less precise measurement and its unobserved truth. We assumed that the gold standard measurements and less precise measurements are conditionally independent given the latent vector (*X*^*EE*^, *X*^Δ*ES*^). We modeled the latent vector (*X*^*EE*^, *X*^Δ*ES*^) using a bivariate normal distribution and a Dirichlet process. Although the Dirichlet process is more flexible and based on a weaker assumption, it required more replicate observations (mainly on gold standard measurements) than is feasible in practice in order to give stable results. The normality assumption was robust and resulted in stable and surprisingly reasonable results given the true structure of the latent variables. Because this model produced accurate estimates even with only two replicates of gold standard measurements per person, we believe that it is a plausibly useful model for this specific application unless more than two replicates per person are available. The resulting estimates and PMSE show the approach what we propose outperforms a simpler linear measurement error model and a naïve model that does not take measurement error into consideration.

The intended use of the model presented in this paper is for device calibration. In order to do meaningful research in the fields of physical activity, nutrition, and health, one needs accurate, reliable data. The issue of obesity was highlighted in the introduction, and understanding energy consumed versus energy expended is crucial to understanding the obesity crisis, but collecting data on these quantities is difficult. Because measurements of EE and ΔES from less expensive devices can often include considerable error and bias, these data can lead to erroneous results later in a study. Although gold standard measurements exits for EE and ΔES, they are expensive and it is unreasonable in a large study to administer gold standard measurements to everything in the study. The method presented in this paper provides a statistical approach that allows for flexibility in the relationship between less expensive measurement and truth in order to calibrate less expensive measurements. This way, large studies can administer both gold standard and less expensive measurements to a small subsample, and use the methods presented in this paper to calibrate the less expensive measurements for those who didn’t receive gold standard measurements. This can save time and money for researchers without having to compromise the integrity of the data. One of the uses would be to obtain a corrected estimate of EI, by getting corrected estimates of EE and ΔES and then using the energy balance equation. Although only a simulation study is presented, given a study with the same data structure, estimates of the parameters in the model could be used for future device calibration.

The main motivation for constructing this model was to account for the error and bias in easy to administer measurements in order to calibrate less precise observations. We presented a simple way to do this calibration given an less precise measurement for EE and ΔES and values of gender, BMI, and age. Using a Bayesian approach we are easily able to get a posterior distribution for the mean calibrated estimate which also provides a measure of uncertainty. Our example shows that the calibrated estimate is often an improvement compared to the observed less precise measurement.

## Supporting information

S1 Appendix Gibbs algorithm for spline model. (PDF)

S2 Appendix Reversible jump MCMC. (PDF)

S3 Appendix Full model likelihood. (PDF)

S4 Appendix Prior distributions. (PDF)
