# Supplementary material for: Modeling energy balance while correcting for measurement error via free knot splines
Source: PLoS One. 2018 Aug 30;13(8):e0201892. doi: 10.1371/journal.pone.0201892 (PMC6116982; doi:10.1371/journal.pone.0201892)
Supplement: S1 Appendix — (PDF) [file pone.0201892.s001.pdf]

S1 Appendix Gibbs Algorithm for the Spline Model. Here we present our Gibbs algorithm to sample from the joint posterior distribution.

1. Set starting values. Let $i = 1, ..., n$ index individuals, and $j = 1, ..., J$ index replicates within individual. Assume for simplicity each individual has the same number of replicates.

2. Run the MCMC below B times. Every b iterations, update tuning parameters C_{X_i}, the covariance for the random walk proposal distribution by setting

$$C_{X_i} = \frac{2.4^2}{2}\frac{1}{B-1}\sum_{b=1}^{B}(X_i^{EE(b)} - \bar{X}^{EE})(X_i^{\Delta ES(b)} - \bar{X}^{\Delta ES}). \quad (1)$$

   Repeat desired number of times, then discard burn-in draws and continue using new tuning values. (See [1], pg. 290)

3. For iteration k=1,...,K, sample from the full conditional distributions:

   (a) $\{\zeta_i^{(k)} : i = 1, ..., n\} \mid \cdot$ where $\mathrm{P}(\zeta_i^{(k)} = h) = \frac{\pi_h^{(k-1)} N(\mu_h^{(k-1)}, \Sigma_h^{(k-1)})}{\sum_{h=1}^{H} \pi_h^{(k-1)} N(\mu_h^{(k-1)}, \Sigma_h^{(k-1)})}$.

   (b) Draw $\{V_h^{(k)} : h = 1, ..., H-1\} |\cdot \overset{ind}{\sim} Beta(1 + n_h, \alpha + n'_h)$,
   With $V_H^{(k)} = 1$
   $n_h = \sum_{i=1}^{n} I(\zeta_i^{(k)} = h)$
   $n'_h = \sum_{h'=h+1}^{H} n_{h'}$.

   (c) Calculate $\{\pi_i^{(k)} : i = 1, ..., n\} = V_i^{(k)} \prod_{\ell<i}(1 - V_\ell^{(k)})$.

   (d) Draw $\{\Sigma_h^{(k)}, h = 1, ..., H\} \mid\cdot \overset{ind}{\sim}$
   $Inv - Wish(d + n_h, \psi + (\mathbf{X_{i,h}^{(k-1)}} - \mu_\mathbf{h}^{(\mathbf{k-1})})'(\mathbf{X_{i,h}^{(k-1)}} - \mu_\mathbf{h}^{(\mathbf{k-1})}))$,
   with
   $\mathbf{X_{i,h}} \equiv \mathbf{X_i} I(\zeta_i^{(k)} = h)$
   $\mathbf{X_i} = (X_i^{EE}, X_i^{\Delta ES})$
   $\mu_\mathbf{h} = (\mu_{EE,h}, \mu_{\Delta ES,h})$.

   (e) Draw $\{(\mu_{EE,h}^{(k)}, \mu_{\Delta ES,h}^{(k)}), h = 1, ..., H\} \mid\cdot \overset{ind}{\sim} N(M'_\mu, C'_\mu)$,
   with
   $C'_\mu = (C_\mu^{-1} + n_h \Sigma_h^{-1(k)})^{-1}$
   $M'_\mu = C'_\mu(C_\mu^{-1} M + n_h \Sigma_h^{-1(k)} \bar{\mathbf{X}}^{(\mathbf{k-1})})$
   $\bar{\mathbf{X}} = \frac{1}{n_h}\sum_{i=1}^{n} \mathbf{X_{i,h}}$.

   (f) Update $\mathbf{X_i}$ with a random walk
   $\{\mathbf{X_i^{(k)}} : i = 1, ..., n\}|\cdot$ for $i = 1, ...n$ sample $\mathbf{X_i^*}$ from $N(\mathbf{X_i^{(k)}}, C_{X_i})$,
   and set $\mathbf{X_i^{(k)}} = \mathbf{X_i^*}$ with probability α_{X_i}, otherwise set $\mathbf{X_i^{(k)}} = \mathbf{X_i^{(k-1)}}$,
   where
   $\alpha_{X_i} = min\left(1, \frac{f(\mathbf{X_i^*}|\cdot)}{f(\mathbf{X_i^{(k-1)}}|\cdot)}\right)$.

   (g) Update $\boldsymbol{\beta_{ee}}, \boldsymbol{\beta_{es}}, k_{ee}, k_{es}, r_{ee}, r_{es}, \boldsymbol{\gamma_{ee}}, \boldsymbol{\gamma_{es}}$ using RJMCMC described in next section. Calculate $s_{ee}(\mathbf{X^{EE(k)}}; \boldsymbol{\beta_{ee}^{(k)}})$ and $s_{es}(\mathbf{X^{\Delta ES(k)}}; \boldsymbol{\beta_{es}^{(k)}})$.

   (h) Draw $\sigma_{\epsilon^{EE}}^{2(k)}|\cdot \sim$
   $IG\left(a_{yee} + J \times \frac{n}{2}, b_{yee} + \frac{1}{2}\sum_{i=1}^{n}\sum_{j=1}^{J}\left(Y_{ij}^{EE} - s_{ee}(X_i^{EE(k)}; \boldsymbol{\beta_{ee}^{(k)}}) - \boldsymbol{\gamma_{ee}}^{(k)'} Z_i\right)^2\right)$.

(i) Draw $\sigma^{2(k)}_{\epsilon^{\Delta ES}}|\cdot \sim$
$IG\left(a_{yes} + J \times \frac{n}{2}, b_{yes} + \frac{1}{2}\sum_{i=1}^{n}\sum_{j=1}^{J}\left(Y_{ij}^{\Delta ES} - s_{es}(X_i^{\Delta ES(k)}; \boldsymbol{\beta_{es}^{(k)}}) - \boldsymbol{\gamma_{es}}^{(k)\prime} Z_i\right)^2\right)$

(j) Draw
$\sigma^{2(k)}_{\nu^{EE}}|\cdot \sim IG\left(a_{wee} + J \times \frac{n}{2}, b_{wee} + \frac{1}{2}\sum_{i=1}^{n}\sum_{j=1}^{J}\left(W_{ij}^{EE} - X_i^{EE(k)}\right)^2\right)$.

(k) Draw
$\sigma^{2(k)}_{\nu^{\Delta ES}}|\cdot \sim IG\left(a_{wes} + J \times \frac{n}{2}, b_{wes} + \frac{1}{2}\sum_{i=1}^{n}\sum_{j=1}^{J}\left(W_{ij}^{\Delta ES} - X_i^{\Delta ES(k)}\right)^2\right)$.
The steps above describe one iteration in the algorithm.

References

1. Gelman A, Carlin JB, Stern HS, Dunson DB, Vehtari A, Rubin DB. Bayesian Data Analysis. Chapman and Hall/CRC; 2014.
