# Supplementary material for: Modeling energy balance while correcting for measurement error via free knot splines
Source: PLoS One. 2018 Aug 30;13(8):e0201892. doi: 10.1371/journal.pone.0201892 (PMC6116982; doi:10.1371/journal.pone.0201892)
Supplement: S2 Appendix — (PDF) [file pone.0201892.s002.pdf]

S2 Appendix Reversible Jump MCMC.

[3] introduced RJMCMC as a means to jump between parameter spaces that have different dimensions within an MCMC algorithm. There have been a number of different approaches to Bayesian estimation of free knot splines using RJMCMC [4], [5], [6], [7]. For the most part, we adopt the approach of Denison et al. This approach performs well relative to the fully Bayesian approach of DiMatteo et al. for smooth and not highly complex functions when an appropriate adjustment is made (that was pointed out in DiMatteo et al.). We do not expect the mean function in our model to be highly complex, so the approach of Denison et al. is likely to perform well in our problem.

Consider the situation where we wish to estimate a regression spline in the following setup

$$\begin{aligned} y_i &= f(x_i; \boldsymbol{\beta}) + \epsilon_i, \\ \epsilon_i &\overset{iid}{\sim} N(0, \sigma^2). \end{aligned} \tag{1}$$

where the $y_i, i = 1, ...n$ are the response variable and $x_i, i = 1, ...n$ is the observed covariate. We do not want to specify the number or location of the knots, but rather estimate them from the data. Under the specifications of Denison et al., in RJMCMC for free knot splines there are three possible transitions:

1. Birth of a knot
2. Death of a knot
3. Movement of a knot,

where in either of the first two transitions the dimension of the parameter space changes. The transition that is proposed depends upon the prior for the number of knots k. Using the notation of Denison et al., let the prior probability for k knots be $p(k)$. Then the probability of attempting a birth step, death step or move step are b_k, d_k, η_k, respectively, where $\eta_k = 1 - b_k - d_k$, and

$$\begin{aligned} b_k &= c \times min\left(1, \frac{p(k+1)}{p(k)}\right) \\ d_k &= c \times min\left(1, \frac{p(k)}{p(k+1)}\right), \end{aligned} \tag{2}$$

where $0 \leq c \leq 1/2$ to ensure $b_k + d_k \leq 1$. If the birth step transition is chosen, a new knot is proposed from the set of available new knots. Denison et al. puts a discrete uniform prior on knot locations so that only observed locations can be knot locations. A proposed new knot is chosen at random from the set $\mathcal{A}$=$\{x_i, i = 1, ...n : x_i$ is not currently a knot or within $\ell + 1$ locations of a current knot$\}$. Here, ℓ is the order of the splines, so in our case ℓ =3. If the death step transition is chosen, an existing knot is picked at random and removed. If the movement transition step is chosen, a current knot is picked at random and moved to a new location at random from the set $\mathcal{A}$. Once new knots are chosen, we construct a spline basis matrix using the observed locations $\{x_i, i = 1, ...n\}$ and the positions of the knots. We then use OLS to estimate the spline parameters $\boldsymbol{\beta}$. Although we could place priors on the spline regression parameters, this adds significant computational burden and results have been similar when comparing estimation with OLS versus fully Bayesian estimation as long as functions are smooth and not highly complex [7], [4]. The acceptance probability for each step is of the form given by [3]:

$$\alpha = min(1, \text{likelihood ratio} \times \text{prior ratio} \times \text{proposal ratio}). \tag{3}$$

Denison et al. give simplified acceptance probabilities for each of the three transitions under a Poisson prior for number of knots and a discrete uniform prior for knot locations. In the regression setup in Denison et al., they then sample variance components using a Gibbs step.

The model that was fitted in [7] is simpler than our model (1) in three respects: (i) our regression model is part of a larger hierarchical model, (ii) we have an additional linear component in the mean function, (iii) the covariate value x_i is a latent variable . The first issue does not present much of a problem thanks to conditional independence assumptions. For issue (ii) we propose to update the linear coefficient parameters at the same time as we update the spline coefficient parameters, using OLS. We understand that this is not a fully Bayesian approach, but we anticipate results to be similar with much less computational burden.

Issue (iii) is not as simple. In the free knot spline setup in Denison et al., they have a regression model of the form (1) where y is the observed response and x is the observed covariate. In our case we do not observe x; rather, it is a latent variable that we draw from its full conditional distribution using the Gibbs algorithm described in Section 2.5.1. Because we sample the x's via the Gibbs algorithm, the basis matrix has to be adjusted in every iteration. We calculate the basis matrix using the current value of the latent variables and the proposed knots. If we accept the proposal, then the knots, spline coefficients, and consequently the estimate of $s_{\cdot}(X^{\cdot(k)}, \boldsymbol{\beta}_{\cdot}^{(k)})$ are all updated. The challenge is what to do when we reject the candidate draw. It seems reasonable to keep the current knot locations and the current spline regression parameters, but keeping the current predictions of $s_{\cdot}(X^{\cdot(k)}, \boldsymbol{\beta}_{\cdot}^{(k)})$ does not make sense because they are based on $X^{\cdot(k)}$, which is updated in every iteration. We propose the following protocol: if we reject the RJ candidate, we keep the current knots and spline coefficients, calculate a new basis matrix of X based on the current values of $X^{\cdot(k)}$ and the current knots, and then compute the predicted values by multiplying the basis matrix by the current spline coefficients. An additional complication is that X has to do with the birth step and the set of available knots $\mathcal{A}$ from which to choose . We want to pick randomly from the set of X values which we drew in the current iteration of the MCMC, but we do not want a knot to be bigger than or smaller than the maximum or minimum value of X. To avoid this problem, we exclude the three smallest and the three largest values of X from $\mathcal{A}$. We do not expect this to be a major constraint because we are less concerned with flexibility of the function at its extremes and therefore do not want to allow lots of knots at the extremes.

This algorithm is run independently for EE and ΔES mean regression functions because of the conditional independence assumptions. Because this algorithm is the same for EE and ΔES, we omit subscripts and superscripts below. The reversible jump step that goes into our overall Gibbs algorithm is then:

1. Calculate b_k and d_k according to (2).
2. Select birth, death, or move step with probabilities $b_k, d_k, 1 - b_k - d_k$ respectively.
3. **Knot Changes**
   If birth step:
   (a) Select a new knot location at random from the set $\mathcal{A}$ and join with current knots $r^{(k-1)}$ to create the proposed knot locations r^*.
   If death step:

(b) Sample one knot location from $r^{(k-1)}$ at random and remove it to create the proposed knot locations r^*.

If move step:

(c) Sample one knot location from $r^{(k-1)}$ at random, and change it to a new knot location at random from the set $\mathcal{A}$ to create the proposed knot locations r^*.

4. Calculate the spline basis matrix $B^*(\mathbf{X^{(k)}})$ using $\mathbf{X^{(k)}}$ and proposed knot locations r^*.

5. Calculate proposed spline and linear regression coefficients $\boldsymbol{\beta^*}, \boldsymbol{\gamma^*}$ by using OLS to regress $\bar{\mathbf{Y}}$ on $B^*(\mathbf{X^{\cdot(k)}}) + \mathbf{Z}$
$\bar{\mathbf{Y}} = \left\{ \frac{1}{J} \sum_{j=1}^{J} Y_{ij} : i = 1, ..., n \right\}$.

6. Accept proposed knots and coefficients with probability α, with α shown below:

$$\alpha_{birth} = min\left(1, \text{Likelihood ratio} \times \frac{n - Z(k)}{n}\right),$$
$$\alpha_{death} = min\left(1, \text{Likelihood ratio} \times \frac{n}{n - Z(k)}\right),$$
$$\alpha_{move} = min\left(1, \text{Likelihood ratio}\right),$$

Otherwise set $r^{(k)} = r^{(k-1)}$, $\boldsymbol{\beta^{(k)}} = \boldsymbol{\beta^{(k-1)}}$, $\boldsymbol{\gamma^{(k)}} = \boldsymbol{\gamma^{(k-1)}}$, and

$$Z(k) = 2(\ell + 1) + k.(2\ell + 1),$$
$$k = length(r^{(k-1)}).$$

7. Compute mean function $s(\mathbf{X^{(k)}}; \boldsymbol{\beta^{(k)}})$ using spline basis matrix $B'(\mathbf{X^{(k)}})$ and $r^{(k)}$

References

1. Bouten C, Verboeket-van de Venne W, Westerterp KR, Verduin M, Janssen JD. Daily physical activity assessment: comparison between movement registration and doubly labeled water. Journal of Applied Physiology. 1996;81:1019–26.

2. Fernandez C, Steel MFJ. On Bayesian Modeling of Fat Tails and Skewness. Journal of the American Statistical Association. 1998;93:359–71.

3. Green PJ. Reversible Jump Markov Chain Monte Carlo Computation and and Bayesian Model Determination. Biometrika. 1995;82:711–32.

4. DiMatteo I, Genovese CR, Kass RE. Bayesian Curve-Fitting with Free-Knot Splines. Biometrika. 2001;88:1055–71.

5. Lindstrom MJ. Bayesian estimation of free-knot splines using reversible jumps. Computational Statistics and Data Analysis. 2002;41:255–69.

6. Johnson MS. Modeling dichotomous item responses with free-knot splines. Computational Statistics and Data Analysis. 2007;51:4178–92.

7. Denison DGT, Mallick BK, Smith AFM. Automatic Bayesian curve fitting. Journal of the Royal Statistical Society. 1998;60:333–50.
