# Supplementary material for: Modeling energy balance while correcting for measurement error via free knot splines
Source: PLoS One. 2018 Aug 30;13(8):e0201892. doi: 10.1371/journal.pone.0201892 (PMC6116982; doi:10.1371/journal.pone.0201892)
Supplement: S3 Appendix — (PDF) [file pone.0201892.s003.pdf]

S3 Appendix Full Model Likelihood

$$
\begin{aligned}
L_i(\boldsymbol{\theta}) &= \prod_{j=1}^{J} f(W_{ij}^{EE}, W_{ij}^{\Delta ES}, Y_{ij}^{EE}, Y_{ij}^{\Delta ES} | Z_i, \boldsymbol{\theta}) \\
&= \int_{\mathcal{X}_{es}} \int_{\mathcal{X}_{ee}} \prod_{j=1}^{J} f(W_{ij}^{EE}, W_{ij}^{\Delta ES}, Y_{ij}^{EE}, Y_{ij}^{\Delta ES}, X_i^{EE}, X_i^{\Delta ES} | Z_i, \boldsymbol{\theta}) dX_i^{EE} dX_i^{\Delta ES} \\
&= \prod_{j=1}^{J} \int_{\mathcal{X}_{es}} \int_{\mathcal{X}_{ee}} f(W_{ij}^{EE} | X_i^{EE}, X_i^{\Delta ES}, Z_i, \boldsymbol{\theta_{wee}}) f(W_{ij}^{\Delta ES} | X_i^{EE}, X_i^{\Delta ES}, Z_i, \boldsymbol{\theta_{wes}}) \times \\
& f(Y_{ij}^{EE} | X_i^{EE}, X_i^{\Delta ES}, Z_i, \boldsymbol{\theta_{yee}}) f(Y_{ij}^{\Delta ES} | X_i^{EE}, X_i^{\Delta ES}, Z_i, \boldsymbol{\theta_{yes}}) \times \\
& f(X_i^{EE}, X_i^{\Delta ES} | Z_i, \boldsymbol{\theta_x}) dX_i^{EE} dX_i^{\Delta ES} \\
&= \prod_{j=1}^{J} \int_{\mathcal{X}_{es}} \int_{\mathcal{X}_{ee}} f(W_{ij}^{EE} | X_i^{EE}, Z_i, \boldsymbol{\theta_{wee}}) f(W_{ij}^{\Delta ES} | X_i^{\Delta ES}, Z_i, \boldsymbol{\theta_{wes}}) \times \\
& f(Y_{ij}^{EE} | X_i^{EE}, Z_i, \boldsymbol{\theta_{yee}}) f(Y_{ij}^{\Delta ES} | X_i^{\Delta ES}, Z_i, \boldsymbol{\theta_{yes}}) \times \\
& f(X_i^{EE}, X_i^{\Delta ES} | Z_i, \boldsymbol{\theta_x}) dX_i^{EE} dX_i^{\Delta ES},
\end{aligned}
\tag{1}
$$

where the RHS is expanded in (1). Since we assume that individuals are independent, the full likelihood is

$$
L(\boldsymbol{\theta}) = \prod_{i=1}^{n} L_i(\boldsymbol{\theta}). \tag{2}
$$
