# Supplementary material for: Modeling energy balance while correcting for measurement error via free knot splines
Source: PLoS One. 2018 Aug 30;13(8):e0201892. doi: 10.1371/journal.pone.0201892 (PMC6116982; doi:10.1371/journal.pone.0201892)
Supplement: S4 Appendix — (PDF) [file pone.0201892.s004.pdf]

S4 Appendix Prior Distributions

1. Prior Distributions for Naïve Model

$$\beta_{0,ee} \sim N(M_{\beta_{0,ee}}, C_{\beta_{0,ee}}) \tag{1}$$
$$\beta_{1,ee} \sim N(M_{\beta_{1,ee}}, C_{\beta_{1,ee}}) \tag{2}$$
$$\gamma_{g,ee} \sim N(M_{\gamma_{ee}}, C_{\gamma_{ee}}),\ g = 1, 2, 3 \tag{3}$$
$$\sigma^2_{\epsilon^{ee}} \sim IG(a_{yee}, b_{yee}) \tag{4}$$
$$\beta_{0,es} \sim N(M_{\beta_{0,es}}, C_{\beta_{0,es}}) \tag{5}$$
$$\beta_{1,es} \sim N(M_{\beta_{1,es}}, C_{\beta_{1,es}}) \tag{6}$$
$$\gamma_{g,es} \sim N(M_{\gamma_{es}}, C_{\gamma_{es}}),\ g = 1, 2, 3 \tag{7}$$
$$\sigma^2_{\epsilon^{es}} \sim IG(a_{yes}, b_{yes}). \tag{8}$$

IG refers to the inverse gamma distribution, and $\sigma^2_{\epsilon^{ee}}$ and $\sigma^2_{\epsilon^{es}}$ represent the measurement error and within-person variability as measured in the less precise EE and ΔES tools, respectively.

2. Prior Distributions for LMEM

$$\beta_{0,ee} \sim N(M_{\beta_{0,ee}}, C_{\beta_{0,ee}}) \tag{9}$$
$$\beta_{1,ee} \sim N(M_{\beta_{1,ee}}, C_{\beta_{1,ee}}) \tag{10}$$
$$\gamma_{g,ee} \sim N(M_{\gamma_{ee}}, C_{\gamma_{ee}}),\ g = 1, 2, 3 \tag{11}$$
$$\sigma^2_{\epsilon^{ee}} \sim IG(a_{yee}, b_{yee}) \tag{12}$$
$$\sigma^2_{\nu^{ee}} \sim IG(a_{wee}, b_{wee}) \tag{13}$$
$$\Sigma_X \sim Inv - Wish(\psi, d) \tag{14}$$
$$\beta_{0,es} \sim N(M_{\beta_{0,es}}, C_{\beta_{0,es}}) \tag{15}$$
$$\beta_{1,es} \sim N(M_{\beta_{1,es}}, C_{\beta_{1,es}}) \tag{16}$$
$$\gamma_{g,es} \sim N(M_{\gamma_{es}}, C_{\gamma_{es}}),\ g = 1, 2, 3 \tag{17}$$
$$\sigma^2_{\epsilon^{es}} \sim IG(a_{yes}, b_{yes}) \tag{18}$$
$$\sigma^2_{\nu^{es}} \sim IG(a_{wes}, b_{wes}) \tag{19}$$
$$\mu_{EE}, \mu_{\Delta ES} \sim N(M_\mu, C_\mu). \tag{20}$$

3. Prior Distributions for SMEM

$$\sigma^2_{\epsilon^{ee}} \sim IG(a_{yee}, b_{yee}) \tag{21}$$
$$\sigma^2_{\nu^{ee}} \sim IG(a_{yes}, b_{yes}) \tag{22}$$
$$k_{ee} \sim Poi(\lambda_{ee}) \tag{23}$$
$$r_{ee} \sim DisUnif(X_1^{EE}, ..., X_n^{EE}) \tag{24}$$
$$\Sigma_h \sim Inv - Wish(\psi, d), h = 1, ...H \tag{25}$$
$$\sigma^2_{\epsilon^{es}} \sim IG(a_{wee}, b_{wee}) \tag{26}$$
$$\sigma^2_{\nu^{es}} \sim IG(a_{wes}, b_{wes}) \tag{27}$$
$$k_{es} \sim Poi(\lambda_{es}) \tag{28}$$
$$r_{es} \sim DisUnif(X_1^{\Delta ES}, ..., X_n^{\Delta ES}) \tag{29}$$
$$\mu_{EE,h}, \mu_{\Delta ES,h} \sim N(M_\mu, C_\mu), h = 1, ...H. \tag{30}$$
